# Entanglement-induced collective many-body interference

**DOI:** 10.1126/sciadv.adp9030

**Published:** 2024-08-30

**Authors:** Tommaso Faleo, Eric Brunner, Jonathan W. Webb, Alexander Pickston, Joseph Ho, Gregor Weihs, Andreas Buchleitner, Christoph Dittel, Gabriel Dufour, Alessandro Fedrizzi, Robert Keil

**Affiliations:** ^1^ Institut für Experimentalphysik, Universität Innsbruck, Technikerstr. 25, 6020 Innsbruck, Austria.; ^2^ Physikalisches Institut, Albert-Ludwigs-Universität Freiburg, Hermann-Herder-Straße 3, 79104 Freiburg, Germany.; ^3^ Institute of Photonics and Quantum Sciences, School of Engineering and Physical Sciences, Heriot-Watt University, Edinburgh EH14 4AS, UK.; ^4^ EUCOR Centre for Quantum Science and Quantum Computing, Albert-Ludwigs-Universität Freiburg, Hermann-Herder-Straße 3, 79104 Freiburg, Germany.; ^5^ Freiburg Institute for Advanced Studies, Albert-Ludwigs-Universität Freiburg, Albertstraße 19, 79104 Freiburg, Germany.

## Abstract

Entanglement and interference are both hallmark effects of quantum physics. Particularly rich dynamics arise when multiple (at least partially) indistinguishable particles are subjected to either of these phenomena. By combining both entanglement and many-particle interference, we propose an interferometric setting through which *N*-particle interference can be observed, while any interference of lower orders is strictly suppressed. We experimentally demonstrate this effect in a four-photon interferometer, where the interference is nonlocal, in principle, as only pairs of photons interfere at two separate and independent beam splitters. A joint detection of all four photons identifies a high-visibility interference pattern varying as a function of their collective four-particle phase, a genuine four-body property.

## Teaser

Entanglement of subsets of particles at the input ports of independent interferometers unveils nonlocal collective dynamics.

## INTRODUCTION

Entanglement is arguably one of the most fascinating and powerful phenomena arising in quantum physics. If two or more quantum objects are entangled, then they can no longer be described as independent entities; instead, their properties are interlinked through their associated degrees of freedom (dof), regardless of how these dof are measured and how far apart the objects are from each other (*1*). This effect has been famously and unambiguously demonstrated in Bell inequality tests (*2*, *3*), and it enables a rich variety of applications within the fields of quantum communication (*4*–*6*), quantum computation (*7*–*9*), and quantum simulation (*10*, *11*).

A similarly fundamental effect is given by two-particle (2P) interference, as first observed by Hong, Ou, and Mandel (HOM) (*12*) via the absence of photon pair coincidences at the output of a balanced beam splitter. HOM interference has emerged as one of the most widely used quantum effects in the field of photonics (*13*), and it requires indistinguishability of the involved particles. Specifically, particles must not be identifiable by their internal states, the states of those dof that do not participate in the dynamics, thus forbidding the retrieval of 2P which-way information (*14*, *15*). The HOM experiment represents a starting point for the exploration of multiparticle interference phenomena, and its generalization to larger-scale *N*-particle (*N*P) systems (*16*–*18*) finds applications in fundamental tests of quantum mechanics (*19*) and in the advancement of quantum technologies (*20*, *21*). However, *N*P interference for *N* > 2 is inherently more complex than the HOM effect. The rich spectrum of multiparticle interference terms stemming from the various particle-exchange processes gives rise to nontrivial behaviors, especially when partial distinguishability among the particles is introduced (*18*, *22*, *23*). Therefore, a substantial challenge is to discern genuine *N*P interference (not reducible to independent interference processes of 2 ≤ *m* < *N* particles), which has, in particular, been tackled by examining specific signatures of this interference (*24*–*28**)* and by identifying instances of totally destructive *N*P interference, which generalize the HOM scenario (*17*, *29*–*32*). These destructive *N*P interferences arise from the cancellation of many-particle transition amplitudes in the complex plane, reminiscent of selective excitations of *N*-quanta transitions in nuclear magnetic resonance (*33*). Recent experimental observations (*34*, *35*) have, furthermore, demonstrated the presence of genuine multiparticle interference witnessed by its dependence on a collective geometric phase that is determined by the internal quantum state of all *N* particles. The emergence of this collective *N*P phase can be attributed to carefully engineer internal states of the particles, as well as the specific structure of the interference terms, involving cyclic permutations of all *N* particles (*36*).

Here, we propose and experimentally demonstrate an interference effect to realize genuine *N*P interference by combining the concepts of entanglement and many-body interference. In particular, we show that, by using entanglement between a subset of particles at the input of separate interferometers, full-contrast interference fringes emerge for the *N*P correlator as a function of a collective *N*P phase, while the signals of all lower-order correlators remain independent of the involved phases. In contrast to the realization of *N*P phases of previous approaches (*34*, *35*), which necessitate the engineering of an *N*-dimensional internal Hilbert space and the mixing of all particles in an *N*-cyclic interferometer, here, only a two-dimensional internal Hilbert space and pairwise exchanges of particles at separate beam splitters are needed to induce collective *N*P interference.

By virtue of its design, the entanglement-induced collective *N*P interference effect presented here exhibits full interference contrast for arbitrarily large numbers of particles and is, by construction, independent of the spatial separation between the beam splitters, such that the resulting collective many-body interference can be realized in a nonlocal fashion. Multiparticle interference in disjoint interferometers is otherwise only known from Franson-interference (*37*, *38*), which relies on maximally entangled states in the energy-time dof intrinsically linked to the photon generation process. In contrast, the entanglement-induced collective phase shown in this work can be imprinted on any bosonic state with a two-dimensional internal Hilbert space.

## RESULTS

### Many-body interference and collective phases

**Fig. 1.**
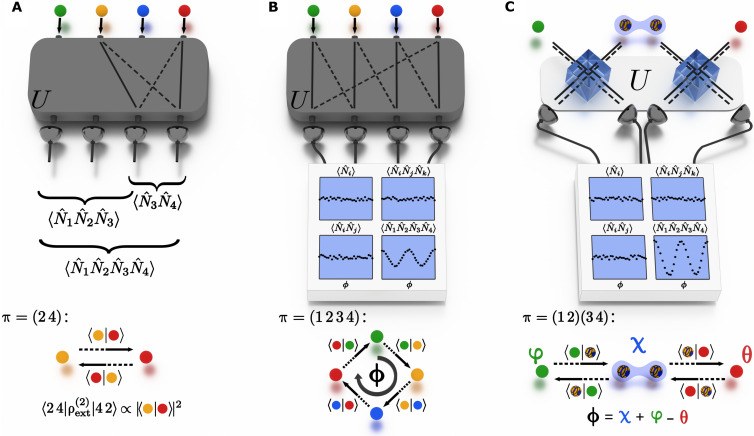
Many-particle interference and collective phases. (**A**) In a fully connected interferometer with single-particle unitary matrix *U* (here 4 × 4), multiple exchange processes among combinations of input-output channels contribute to specific output events. The reduced 2P state associated with the second (yellow) and the fourth (red) particles contributes to the two-point correlator 〈*N*_3_*N*_4_〉 via the 2P transitions indicated by the solid and dashed lines. The interference of these two 2P paths is encoded in the 2P coherence at the bottom of the panel, which is associated with the permutation π = (1 2) obtained by taking one 2P path in the forward direction (dashed lines) followed by the other one in the backward direction (solid lines) (*60*). The matrix elements of *U* weigh these contributions, as well as the contributions from all other 2P sets. Similar considerations apply to all correlators from combinations of different output modes. (**B**) The four-particle (4P) coherence corresponding to the depicted four-cyclic permutation leads to a genuine 4P interference that depends on a collective phase ϕ. This collective phase is set by the summation of phases ϕ*_ij_* (see section on Many-body interference and collective phases), resulting from the overlaps of the particles’ internal states along a “circle-dance” graph representative of the permutation process (*35*, *36*), as depicted at the bottom of the panel. (**C**) A genuine entanglement-induced 4P interference can be achieved by interfering entangled particles (blue envelope) with particles in separable states in two independent and separate beam splitters. In this process, the entanglement induces a collective 4P phase term ϕ, set by the internal states of all particles, through the two 2P permutations in π = (1 2)(3 4). The collective phase ϕ only affects the four-point correlator and introduces full-contrast interference fringes: 〈*N*_1_*N*_2_*N*_3_*N*_4_〉 ∝ cos^2^(ϕ/2) (see section on Scheme for entanglement-induced collective interference).

In a general interference scenario among *N* particles, as depicted in Fig. 1A for *N* = 4 unentangled particles, each input mode *m_i_* = 1, …, *N* of the multiport interferometer is occupied by a single particle, which carries some internal dof (illustrated by the particle’s color in Fig. 1). Interference effects across the multiport manifest through correlations of the particle numbers at the output ports of the interferometer. We specifically consider correlations between the occupations of *k* ≤ *N* distinct modes *p*_1_, …, *p_k_*. Denoting by *U* the single-particle unitary matrix describing the transformation between input and output modes (henceforth termed external modes) implemented by the interferometer, the expectation value of such a *k*-point correlator is given by

$$\langle N_{p_{1}}\ldots N_{p_{k}}\rangle =\frac{N!}{\left(N-k\right)!}\sum_{m,n}‍\prod_{i=1}^{k}‍U_{p_{i}m_{i}}U_{p_{i}n_{i}}^{*}\left\langle m\left|\rho_{\text{ext}}^{\left(k\right)}\right|n\right\rangle$$ (1)

where the external number operator *N_p_* counts the number of particles in output mode *p*, irrespective of their internal state. In terms of the operator *a*_*p*α_ annihilating a particle with internal state ∣α⟩ in external mode *p*, the external number operator reads $N_{p}=\sum_{\alpha }‍a_{p\alpha }^{†}a_{p\alpha }$, where the sum runs over a basis of the internal Hilbert space.

Because it involves products of *k* creation and *k* annihilation operators, the *k*-point correlator *N*_*p*_1__…*N_p_k__* is an example of a *k*-particle (*k*P) observable (*27*, *39*, *40*). As a consequence, for *k* < *N*, it only probes the reduced state $\rho_{\text{ext}}^{\left(k\right)}$, obtained by tracing out the internal dof and (*N − k*) particles from the input state (*27*, *41*, *42*). Specifically, the matrix elements $\left\langle m\left|\rho_{\text{ext}}^{\left(k\right)}\right|n\right\rangle$ appear in Eq. 1, with ∣***m***⟩ = ∣ *m*_1_⟩ ⊗ … ⊗ ∣ *m_k_*⟩ being the tensor product of the external single-particle basis states ∣*m_i_*⟩, corresponding to the particle occupying input port *m_i_* of the interferometer, and analogously for ∣***n***⟩. The nonzero matrix elements $\left\langle m\left|\rho_{\text{ext}}^{\left(k\right)}\right|n\right\rangle$ are indexed by tuples ***m*** and ***n*** that are connected by a permutation π ∈ S*_k_* in the symmetric group S*_k_* of their *k* entries: ***n*** = π(***m***) = (*m*_π_^−1^(_1_), …, *m*_π_^−1^(_*k*_)). The off-diagonal matrix elements (***m*** ≠ ***n***) are called *k*P coherences.

In the traditional scenario where each particle in mode *m_i_* is associated with a particular internal quantum state ∣ξ*_m_i__*⟩, corresponding to an input product state $\left|\Psi \rangle =a_{m_{1},\xi_{m_{1}}}^{†}\ldots a_{m_{N},\xi_{m_{N}}}^{†}|0\right\rangle$, all matrix elements are set, up to a multiplicative constant, by the overlaps between the internal states of the particles in the specific *k* modes

$$\langle m\left|\rho_{\text{ext}}^{\left(k\right)}\right|n\rangle \propto \prod_{i=1}^{k}‍\langle \xi_{m_{i}}|\xi_{m_{\pi^{-1}\left(i\right)}}\rangle$$ (2)

Therefore, these overlaps, i.e., the particles’ mutual (in)distinguishabilities, determine all matrix elements. In general, the overlaps are complex numbers $\langle \xi_{i}|\xi_{j}\rangle =r_{ij}e^{{i\phi }_{ij}}$, with real amplitude *r_ij_* and phase ϕ*_ij_*. In the two-point correlator 〈*N*_3_*N*_4_〉 shown in Fig. 1A, for example, the indicated transition processes lead to HOM-type interference involving the diagonal term $\left\langle 2,4\left|\rho_{\text{ext}}^{\left(2\right)}\right|2,4\right\rangle \propto \langle \xi_{2}|\xi_{2}\rangle \langle \xi_{4}|\xi_{4}\rangle =1$, and the 2P coherence $\left\langle 2,4\left|\rho_{\text{ext}}^{\left(2\right)}\right|4,2\right\rangle \propto \langle \xi_{2}|\xi_{4}\rangle \langle \xi_{4}|\xi_{2}\rangle =r_{2,4}^{2}$, corresponding to the permutations [in cycle notation; (*43*)] π=I, the identity, and π = (1 2), respectively (see Fig. 1A, bottom). As in HOM (i.e., 2P) interference, no phase dependence arises, and only the real amplitude $r_{2,4}^{2}$, which expresses the particles’ pairwise indistinguishability, determines 〈*N*_3_*N*_4_〉 (*12*).

In more complex multiparticle exchanges, the engineering of internal states ∣ξ*_i_*⟩ within an *N*-dimensional Hilbert space allows to witness a phase dependence that is set by the specific permutation π and the overlaps among particle pairs (*34*, *35*). Notably, the genuine *N*P interference introduced in (*36*) is rooted in a *N*P coherence $\left\langle m\left|\rho_{\text{ext}}^{\left(N\right)}\right|\pi \left(m\right)\right\rangle$ associated with a *N*-cyclic permutation π = (1 2 3…*N*), as shown in Fig. 1B, which depends on the “circle-dance” collective *N*P phase ϕ = ϕ_1,2_ + ϕ_2,3_ + … + ϕ_*N*,1_. Engineering zero overlaps of the internal states of non-neighboring particles in the permutation, particles in disconnected vertices of the permutation graph, ensures that no other phase dependence appears in the signal. In this traditional collective phase interference scenario, however, control over an (at least) *N*-dimensional internal Hilbert space is required.

### Scheme for entanglement-induced collective interference

We here introduce a scheme using entanglement to witness a collective *N*P phase, depicted for *N* = 4 in Fig. 1C. In this scheme, the interferometer consists of *N*/2 separate beam splitters, where the first one mixes modes 1 and 2, the second one mixes modes 3 and 4, and so on. Pairwise 2P entanglement in one of the internal dof (here polarization) between particles in modes 2 and 3, 4 and 5, …, *N −* 2 and *N −* 1 can then be used to induce collective interference across the separate beam splitters. For simplicity, we now restrict our discussion to *N* = 4 (as realized in our experiment), while the general case of *N* particles (with *N* even) can be found in Supplementary Note 1. As input, we consider the combination of a polarization-entangled photon pair in modes 2 and 3 and an unentangled photon pair in the remaining modes 1 and 4 (see Fig. 1C). This is described by the four-particle (4P) state

$$\left|\psi \rangle =\frac{1}{\sqrt{2}}a_{1,S}^{†}\left(\varphi \right)\left(a_{2,H}^{†}a_{3,V}^{†}+e^{-i\chi }a_{2,V}^{†}a_{3,H}^{†}\right)a_{4,S}^{†}\left(\theta \right)|0\right\rangle$$ (3)

where $a_{i,H\left(V\right)}^{†}$ creates a photon in external mode *i* with horizontal (vertical) polarization internal state, and

$$a_{i,S}^{†}\left(\varphi \right)=\frac{1}{\sqrt{2}}\left(a_{i,H}^{†}+e^{i\varphi }a_{i,V}^{†}\right)$$ (4)

sets the polarization to a balanced superposition in the *H*/*V* basis with phase φ, and analogously for $a_{i,S}^{†}\left(\theta \right)$. Note that, while the expression of the *k*-point correlator in Eq. 1 remains valid, Eq. 2 no longer holds for such entangled input states.

Because of the specific topology of the interferometer in Fig. 1C (defining the single-particle unitary *U*), not all four modes mix, such that many terms in Eq. 1 vanish. The only *k*P coherences that contribute are associated with permutations π generated by transpositions (2*n* − 1 2*n*), with *n* = 1, …, *N*/2, e.g., for *N* = 4, π = (1 2), π = (3 4), and their product π = (1 2)(3 4). This factorization reflects the structure of the interferometer, with two separate beam splitters, which can, in principle, be arbitrarily far apart. If we assume the input to be given only by the first term of the operator-valued sum acting upon ∣0⟩ in Eq. 3, then Eq. 2 holds. Defining the internal state $|\xi \rangle =\left(a_{H}^{†}+e^{i\xi }a_{V}^{†}\right)|0\rangle /\sqrt{2}$, the contributions arising from particle exchanges according to the above three permutations are given by |〈φ ∣ *H*〉|^2^, |〈*V* ∣ θ〉|^2^, and |〈φ ∣ *H*〉|^2^|〈*V* ∣ θ〉|^2^, respectively, i.e., by the modulus square of the inner products of the exchanged particles’ internal states. These are independent of φ, θ, and χ. Analogously, if only the second term on the right-hand side (RHS) of Eq. 3 was considered, then no phase contributions would be present in the four-point correlator (Eq. 1). The phase dependence results from the superposition of both 4P states created on the RHS of Eq. 3. The additional cross terms are the projection of the first term in Eq. 3 onto the second term exchanged according to π = (1 2)(3 4), i.e., the matrix element $\left\langle 1,2,3,4\left|\rho_{\text{ext}}^{\left(4\right)}\right|2,1,4,3\right\rangle$ in Eq. 1, and its complex conjugate thereof. This matrix element is proportional to *e*^−*i*χ^〈φ∣*V*〉〈*V* ∣ θ〉〈θ ∣ *H*〉〈*H* ∣ φ〉 ∝ *e*^−*i*(χ + φ − θ)^. Note that the four bra-vectors in the scalar product are given by a cyclic permutation of the four ket-vectors. Hence, the permutation π = (1 2)(3 4) in combination with the exchange of the roles of modes 2 and 3 in the two terms of the entangled state ∣ψ⟩ (Eq. 3; see also Fig. 1C) can be viewed as an effective four-cycle permutation that acts on the particles’ internal states (see also Eq. 2), which captures the dependence on the complex phases φ, χ, and θ. Overall we find, for *k* = 4

$$\langle N_{1}N_{2}N_{3}N_{4}\rangle =\frac{1}{8}\text{co}s^{2}\left(\frac{\phi }{2}\right)$$ (5)

with collective 4P phase ϕ = χ + φ − θ. The full derivation is given in Supplementary Note 1. Thus, the 4P correlator oscillates with full interference contrast. One can show that the full contrast remains for arbitrary even particle numbers *N* > 4 by adding beam splitters in parallel and entangled pairs between the beam splitters as discussed in the beginning of this section. Specifically, for an *N*P input state, the *N*-point correlator can be generalized to (see Supplementary Note 1)

$$\langle N_{1}\ldots N_{N}\rangle =\frac{1}{2^{N}}\left(1+{\left(-1\right)}^{\frac{N}{2}}\text{cos}\phi \right)$$ (6)

with collective *N*P phase ϕ = χ_1_ + χ_2_ + … + χ_(*N* − 2)/2_ + φ − θ, where χ_1_, χ_2_, …, χ_(*N* − 2)/2_ are the phases associated with each entangled pair.

In contrast, for *k* < *N*, the structure of the input state and the specific interferometer topology ensure that all probed matrix elements $\left\langle m\left|\rho_{\text{ext}}^{\left(k\right)}\right|n\right\rangle$ in the expectation value (Eq. 1) are independent of the phases φ, χ_1_, χ_2_, …, θ. In the 4P scenario, this can be understood as follows: First, note that the three-point correlator only depends on the matrix elements of $\rho_{\text{ext}}^{\left(3\right)}$ (see Eq. 1). Moreover, the considered interferometer does not mix the modes 1 and 2 with the modes 3 and 4, and the only nonvanishing *k*P coherences $\left\langle m\left|\rho_{\text{ext}}^{\left(k\right)}\right|n\right\rangle$ are indexed by *k*-tuples ***m*** and ***n*** that contain the same modes, only differing by a relative permutation ***n*** = π(***m***), as discussed after Eq. 1. Therefore, the only contributing 3P coherences in Eq. 1 for *k* = 3 are, up to complex conjugation and permuting both index tuples by the same permutation, $123\left|\rho_{\text{ext}}^{\left(3\right)}\right|213$, $124\left|\rho_{\text{ext}}^{\left(3\right)}\right|214$, $134\left|\rho_{\text{ext}}^{\left(3\right)}\right|143$, and $234\left|\rho_{\text{ext}}^{\left(3\right)}\right|243$.

In all these terms, only two particles are exchanged, similar to the 2P HOM interference case (see Fig. 1A), which only involves squared moduli of the internal states’ overlaps. For example

$$\langle 123\left|\rho_{\text{ext}}^{\left(3\right)}\right|213\rangle \propto {\left|\langle \varphi |H\rangle \right|}^{2}+{\left|\langle \varphi |V\rangle \right|}^{2}$$ (7)

The first contribution on the RHS comes from the first term in the sum (after multiplying all factors out) on the RHS of Eq. 3 defining the input state. Because the particle in input mode 2 has horizontal polarization, the exchange π = (12) leads to |〈φ∣*H*〉|^2^. Correspondingly, the second contribution on the RHS of Eq. 7 stems from the second term on the RHS of Eq. 3, where the particle in mode 2 has vertical polarization. Note that the cross contribution (which encodes the complex phase dependence for the four-point correlator as discussed before Eq. 5) is zero because the particle in mode 3 has orthogonal polarization states in both terms. Analogously, all other contributing matrix elements in Eq. 1 are independent of the phases φ, θ, and χ. The same holds for *k* = 2. As a consequence, the *N*P collective phase remains invisible in all correlation orders lower than *N*, which is why the description of this effect in terms of independent 2P HOM processes fails.

Measurements of *k*-point correlators, as in Eq. 1, require resolving the photon number at each detector. Most photonic experiments, however, typically use threshold detectors and record coincidence events involving *k* detectors, referred to as *k*-fold coincidences, where at least one photon is detected in each of the output ports *p*_1_, …, *p_k_*. In an *N*P scenario, the *N*-fold coincidence rate is equivalent to the *N*-point correlator, because necessarily only one photon impinges each detector. However, *k*-fold coincidence events with *k* < *N* do not constitute true *k*P observables (as multiple photons may impinge on a single detector unnoticed) and are, in general, garnished by contributions from *l*P terms, with *k* < *l* ≤ *N*, thus exhibiting weak traces of the *N*P collective phase ϕ (see Supplementary Note 2).

### Experimental setup

**Fig. 2.**
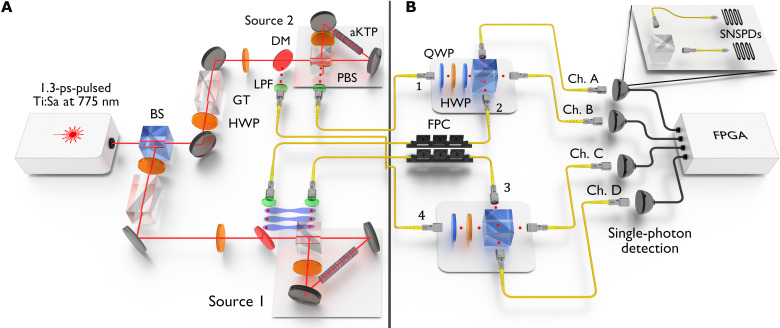
Experimental setup. (**A**) A picosecond (ps)–pulsed laser clocked at 80 MHz is split via a balanced beam splitter (BS) to pump two SPDC sources based on apodized potassium titanyl phosphate (aKTP) crystals embedded in Sagnac interferometers (source 1 and source 2). Half-wave plates (HWPs) and Glan-Taylor polarizers (GT) are used to adjust the input power and set the pump polarization to obtain entangled photon pairs (source 1) or separable photon pairs (source 2). Down-converted photons are separated from the laser light via polarizing beam splitters (PBSs), dichroic mirrors (DMs), long-pass filters (LPFs), and collected through single-mode fibers. (**B**) Photons are adjusted in polarization via combinations of HWPs and quarter-wave plates (QWPs), or via fiber polarization controllers (FPCs) and guided to two independent balanced beam splitters. At the output, photons are detected by multiplexing each output channel (Ch. A to Ch. D) with two SNSPDs at the output ports of a PBS, as shown in the inset. The single-photon detection events are analyzed at a field-programmable gate array (FPGA) logic unit and post-processed to determine every twofold, threefold, and fourfold coincidence event.

We experimentally tested the collective-phase interference as predicted by Eq. 5 by implementing the scheme in Fig. 1C and the input state in Eq. 3. Figure 2 shows the experimental setup. We produce the four-photon input states via two high-brightness, high-purity (spectral purity ≥98%), and high-fidelity telecom (emission wavelength, 1550 nm) spontaneous parametric down-conversion (SPDC) sources with apodized crystals (*44*, *45*), as schematically represented in Fig. 2A. In the used Sagnac interferometer configuration (*46*–*48*), we obtained experimental fidelities of 98.56(2)% and 98.33(2)% when adjusting the phase χ of the entangled photon pair in modes 2 and 3 of Eq. 3 to χ = 0, π (representing the well-known Bell states ∣ψ^+^⟩ and ∣ψ^−^⟩), respectively, as shown in Supplementary Note 3.

Before interfering the photons as shown in Fig. 2B, we prepare the polarization states of the photons in modes 1 and 4 according to Eq. 4, through combinations of quarter- and half-wave plates, and we set the phase χ of the entangled pair via fiber polarization controllers. More precisely, we varied the collective phase of the input state by changing the phase φ of the photons in mode 1 and the phase χ of the entangled pair to obtain the Bell state ∣ψ^+^⟩ or ∣ψ^−^⟩ but fixing the phase θ = 0, corresponding to the setting of a diagonal polarization state $\left|D_{4}\rangle =a_{4,S}^{†}\left(0\right)|0\right\rangle$ of the photons in mode 4.

After interfering, the photons collected at the output ports of the beam splitters are detected via superconducting nanowire single-photon detectors (SNSPDs) with quantum efficiencies of ≥80%. To account for the polarization-dependent SNSPD detection efficiency, we project to the *H/V* basis and multiplex the outputs to eight SNSPD channels. The post-processing of the data retrieves the aggregated single counts of each output channel and all possible combinations of *k*-fold coincidences among these channels, with *k* = 2,3, and 4. The multiplexed eight-detector scheme has two additional advantages: First, because SPDC sources are affected by multi-pair emission contributions (more than two photon pairs produced), this scheme makes it possible to reject coincidences of more than four photons, thus partially cleaning the fourfold statistics from these contributions. Second, the presence of additional beam splitters and (non-number–resolving) detectors helps to provide a pseudo–photon-number resolution (*49*), which is required to perform correlation measurements as in Eq. 1. Although a single multiplexing layer is insufficient for a complete correlation measurement, it already significantly reduces the visibility of the collective phase dependence of any *k*(<4)-fold coincidence events (see example in Supplementary Note 2).

### Experimental results

The specific arrangement of SPDC sources in our experimental scheme causes double-pair emissions from an individual source to propagate through the setup with the same probability as the desired two-source emissions. This results in a fourfold coincidence background from each source with the same order of magnitude as the coincidences from two-source emissions, thus reducing the visibility compared to the expected theoretical prediction of Eq. 5. However, our setup has the advantage of consisting of completely independent beam splitters, such that photons from single-source double-pair emissions do not interfere. The double-pair background is, therefore, independent of the collective phase by construction and can be subtracted from the entanglement-induced four-photon collective interference phase signal. Specifically, by blocking one of the two sources at a time, the four-photon background from the other source can be measured independently of the signal and subtracted from the raw data obtained in the main experiment (*35*). As an alternative to this background subtraction, one can use a three-source scheme, heralding the unentangled photons in modes 1 and 4 to reject the contribution of double emissions from a single source. However, a detailed study of the interference visibility and its uncertainty due to counting statistics shows that the background subtraction solution yields higher visibility and lower visibility error for the same total measurement time.

We recorded photon events for combinations of the two phases χ = 0, π and 31 settings of the phase φ ∈ (−π/2,3π/2), thus testing the collective phase within the 3π phase range ϕ ∈ (−π/2,5π/2). We measured each setting of φ, at fixed χ, over 60 s, and we averaged the measurements over 20 repetitions of this phase scan. We subsequently performed the measurement of the background for each photon source by following the same procedure but using a reduced set of eight phase scans per source to obtain a good trade-off between acquiring sufficient data statistics and avoiding long-term drifts affecting the measurements.

**Fig. 3.**
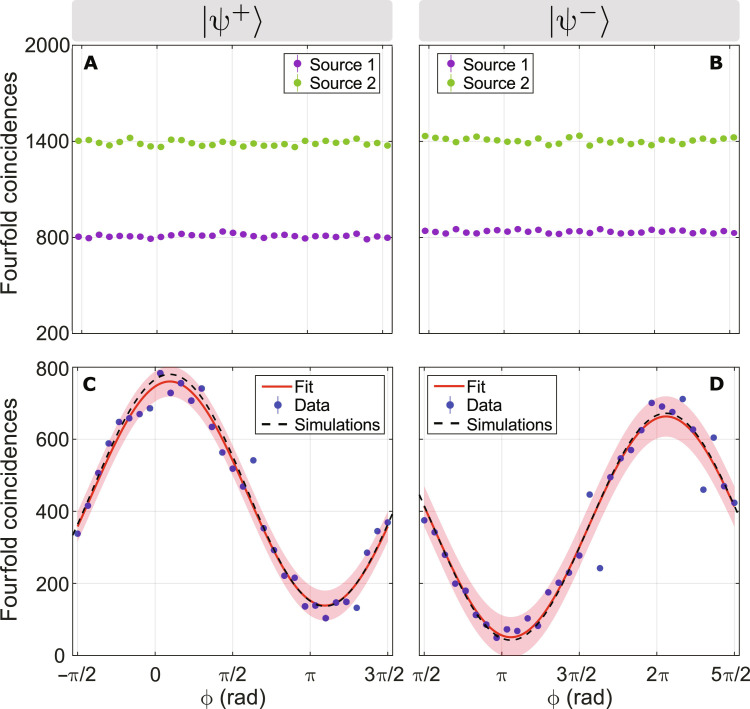
Results of fourfold coincidence counts for entanglement-induced collective 4P interference. (**A** and **B**) Fourfold background coincidences from multi-pair emissions of individual sources. (**C** and **D**) Background-subtracted fourfold coincidences data (blue dots) fitted with a cosine function (red curve). The red-shaded region shows the fit prediction interval at a confidence level of one SD. The graphs include the results of the multiphoton interference simulations (black dashed curve). The visibility of the fit is 69.2(2.5)% and 85.8(4.5)% for (C) and (D), respectively. Simulations (Supplementary Note 5) predict a visibility of 70.2(1.2)% and 88.1(1.7)%. The integration time for each point of all panels is 60 s.

Figure 3 shows the results of both the fourfold background coincidences and the background-subtracted fourfold coincidences when preparing the photon pair in modes 2 and 3 in the two Bell states ∣ψ^+^⟩ (χ = 0) and ∣ψ^−^⟩ (χ = π), respectively. The background coincidences in Fig. 3 (A and B) have negligible fluctuations with relative magnitude ≃1.2%, uncorrelated to the collective phase ϕ. In contrast, the background-subtracted fourfold coincidences in Fig. 3 (C and D) exhibit pronounced cosine oscillations, as predicted by Eq. 5, with high visibilities of 69.2(2.5)% and 85.8(4.5)%, respectively. This distinctive dependence of fourfold coincidences on the collective phase cannot be attributed to fluctuations of single counts or twofold and threefold coincidences, which exhibit only weak fluctuations imputable to secondary effects, as detailed in Supplementary Note 4, where we also present the raw (uncorrected) four-photon coincidence data. Moreover, the experimental data are in good agreement with numerical simulations of the experiment, which provide visibilities of 70.2(1.2)% and 88.1(1.7)%, respectively. The numerical results are obtained by taking into account input and output losses, unbalanced splitting ratios of the beam splitters, multi-pair emissions of the sources up to six photons, and laser power drifts (see Supplementary Note 5).

These numerical simulations also help to quantify the contribution of effects causing the residual visibility reduction of fourfold coincidences in Fig. 3D and, in particular, Fig. 3C. The primary factors affecting the visibility are, in order of significance: laser power drifts, higher-order (>3) photon-pair emissions, and the imperfect implementation of the unitary transformation. Deviations in the average laser power during the main measurement and the two background measurements in Fig. 3 (A and B) lead to the subtraction of a background level that differs from the level affecting the main measurement. The background fourfold coincidences in Fig. 3A are slightly lower than those measured in Fig. 3B. This causes the lower visibility in Fig. 3C with respect to the measurement in Fig. 3D, which is virtually unaffected by power drifts (see simulation in Supplementary Note 5 for details). The second noise contribution is associated with higher-order emissions with at least three photon pairs emitted by the two sources: two pairs in one source and one in the other. Our background subtraction procedure cannot account for this process. Imperfections in the experimental implementation of the unitary transformation are mainly caused by slight manufacturing deviations of beam splitters from a 50:50 splitting ratio. Additional minor contributions include residual polarization dependence of the beam splitters’ splitting ratios, the imperfect spectral purity of the down-converted photons, or slight temporal indistinguishability at the beam splitters, as well as imperfect state preparation.

## DISCUSSION

We have shown in theory and in photonic experiments that the entanglement between particles at the input ports of independent and separate beam splitters sets the stage for a collective interference effect of all particles, which cannot be traced back to the interference of smaller subsets. The peculiarity of this phenomenon lies in the fact that collective many-particle dynamics arises despite the topology of the optical setup inducing only pairwise interference, with some particles even prepared in distinguishable internal states. Entanglement inscribed into the photonic input state bridges the gaps between the disjoint interferometers and, therefore, acts as a mediator of the collective interference in a nonlocal fashion. Compared to the conventional case of separable photons (*35*, *36*), this effect has two fundamental advantages for scaling toward more particles: First, the equivalent interference scenario with *N* > 4 photons can be implemented by adding beam splitters and pairs of entangled photons in parallel, without the requirement of engineering additional internal dof as *N* increases. Thus, the dimension of the single-particle Hilbert space remains constant at two (*41*), with no further increase in the complexity of the state preparation. Second, the visibility of the entanglement-induced collective phase interference is ideally always one and does not diminish with a growing number of particles. Given the characteristic exponential decrease in the (*N*-fold) coincidence probability of many-body interference experiments, shown here in Eq. 6, this advantage will be instrumental in obtaining clear signals in interference scenarios with higher numbers of particles involved.

Our experimental data and numerical simulations produce a high interference visibility, which is mainly limited by the nature of the SPDC photon sources and the errors introduced with the background subtraction. However, for *N* > 4 input particles, the partial (input-output) connectivity of the interferometer necessitates the participation of all *N*/2 sources to achieve an *N*-fold coincidence, that is, background subtraction is no longer required to obtain visibilities comparable with the highest visibility obtained here (∼86%). The detrimental influence of multi-pair emissions could also be completely overcome by using deterministic single-photon sources, such as semiconductor quantum dots, if the required input state can be engineered by appropriate excitation schemes (*50*–*52*) and high photon indistinguishability between separate sources can be achieved (*53*).

Our study gives another notable example of how entanglement can play a key role in shaping interference phenomena (*38*, *54*, *55*). It reveals a hitherto unexplored interference effect that, by virtue of the partial distinguishability of the involved particles, extends the complexity of traditional many-body interference scenarios, potentially leading to unexpected phenomena. For instance, a recent work has shown that by using *N* ≥ 7 partially distinguishable particles, indistinguishable bosons do not maximize the probability of bunching in a subset of output modes (*28*). The scheme presented here allows scaling to such a modest number of interfering particles, raising the question of whether and how a collective behavior, as induced here through entanglement between internal dof of a subset of particles, can affect the dynamics in similar many-body conditions. Moreover, our scheme readily extends to larger entangled Greenberger-Horne-Zeilinger (GHZ) states (*56*) while preserving its properties (see Supplementary Note 1). Using such multipartite entangled states offers various advantages in quantum communication protocols (*57*, *58*). Consequently, it seems worthwhile to further investigate the potential of entanglement-induced collective interference with GHZ states for multiparty quantum communication. Furthermore, the presence of an *N*P collective phase producing high interference contrast could be exploited for quantum metrology purposes. In particular, the possibility of scaling up the scheme by adding only Bell pairs can be beneficial to avoid the typical complex preparation of NOON states and their exposure to decoherence at large photon numbers *N* (*59*). However, given the exponential decrease in the probabilities of individual output coincidence events, the challenge lies in the extraction of information. Overall, the combination of partial distinguishability and entanglement in many-body interference could serve as a valuable tool for advancing quantum technologies or manifest as a subtle yet noteworthy side effect.

## Supplementary Materials

### This PDF file includes:

Supplementary Note S1 to S5 Figs. S1 to S5 References
