# Supplementary material for: Entanglement-induced collective many-body interference
Source: Sci Adv. 2024 Aug 30;10(35):eadp9030. doi: 10.1126/sciadv.adp9030 (PMC11364098; doi:10.1126/sciadv.adp9030)
Supplement: Supplementary file 1 — Supplementary Note S1 to S5 Figs. S1 to S5 References [file sciadv.adp9030_sm.pdf]

Science Advances
AAAS

Supplementary Materials for

Entanglement-induced collective many-body interference

Tommaso Faleo *et al.*

Corresponding author: Tommaso Faleo, tommaso.faleo@uibk.ac.at; Robert Keil, robert.keil@uibk.ac.at

Sci. Adv. **10**, eadp9030 (2024)
DOI: 10.1126/sciadv.adp9030

This PDF file includes:

Supplementary Note S1 to S5
Figs. S1 to S5
References

Supplementary Note 1: N-point correlator and collective phase

We discuss here the dependence of the N-point correlator on the NP collective phase, as shown in Eq. (6) of the main text. The 4-point correlator in Eq. (5) of the main text can be directly derived as a specific case by setting $N = 4$.

For an N-boson state ρ, the reduced k-particle external state $\rho_{\text{ext}}^{(k)}$ is defined by

$$\langle \boldsymbol{m}|\rho_{\text{ext}}^{(k)}|\boldsymbol{n}\rangle = \frac{(N-k)!}{N!}\sum_{\boldsymbol{\alpha}} \text{Tr}(a_{\boldsymbol{m},\boldsymbol{\alpha}}\rho a_{\boldsymbol{n},\boldsymbol{\alpha}}^{\dagger}), \tag{S1}$$

where $\boldsymbol{m} = (m_1, \ldots m_k)$ and $\boldsymbol{n} = (n_1, \ldots n_k)$ are lists of k external modes (input ports of the interferometer), $\boldsymbol{\alpha} = (\alpha_1, \ldots \alpha_k)$ is a list of k internal basis states (two orthogonal polarizations in our case) and $a_{\boldsymbol{m},\boldsymbol{\alpha}} = a_{m_1,\alpha_1}a_{m_2,\alpha_2}\ldots a_{m_k,\alpha_k}$. One goes from the k-particle state of Eq. (S1) to the $k-1$-particle state by setting $n_k = m_k$ and summing over m_k:

$$\langle m_1,\ldots m_{k-1}|\rho_{\text{ext}}^{(k-1)}|n_1,\ldots n_{k-1}\rangle = \sum_{m_k}\langle m_1,\ldots m_{k-1},m_k|\rho_{\text{ext}}^{(k+1)}|n_1,\ldots n_{k-1},m_k\rangle\,. \tag{S2}$$

We let the symmetric group S_k act on lists of k elements by reordering their entries, i.e. for a permutation $\pi \in S_k$,

$$\pi(\boldsymbol{m}) = (m_{\pi^{-1}(1)}, m_{\pi^{-1}(2)}, \ldots m_{\pi^{-1}(k)}). \tag{S3}$$

We then have the invariance property

$$\forall \pi \in S_k, \qquad \langle \pi(\boldsymbol{m})|\rho_{\text{ext}}^{(k)}|\pi(\boldsymbol{n})\rangle = \langle \boldsymbol{m}|\rho_{\text{ext}}^{(k)}|\boldsymbol{n}\rangle\,, \tag{S4}$$

so it is often sufficient to consider matrix elements where $\boldsymbol{m}$ is ordered ($m_1 \leq m_2 \leq \ldots m_k$). If the number of particles per external mode in state ρ is fixed, $\langle \boldsymbol{m}|\rho_{\text{ext}}^{(k)}|\boldsymbol{n}\rangle$ vanishes unless there exists $\pi \in S_k$ such that $\boldsymbol{n} = \pi(\boldsymbol{m})$.

An M-port interferometer is described by the following mapping between the annihilation operators associated with input ($a_{m,\alpha}$) and output ($b_{p,\alpha}$) modes:

$$b_{p,\alpha} = \sum_{p=1}^{M} U_{pm} a_{m,\alpha}, \tag{S5}$$

where U is an $M \times M$ unitary matrix. The correlation between the occupations of k output modes $p_1, p_2, \ldots p_k$ is given by

$$\begin{aligned}\langle N_{p_1} N_{p_2} \ldots N_{p_k} \rangle &= \sum_{\boldsymbol{\alpha}} \mathrm{Tr}(\rho b^\dagger_{\boldsymbol{p},\boldsymbol{\alpha}} b_{\boldsymbol{p},\boldsymbol{\alpha}}) \\ &= \sum_{\boldsymbol{m},\boldsymbol{n}} \sum_{\boldsymbol{\alpha}} \left(\prod_{i=1}^{k} U_{p_i,m_i} U^*_{p_i,n_i} \right) \mathrm{Tr}(\rho a^\dagger_{\boldsymbol{n},\boldsymbol{\alpha}} a_{\boldsymbol{m},\boldsymbol{\alpha}}) \\ &= \frac{N!}{(N-k)!} \sum_{\boldsymbol{m},\boldsymbol{n}} \left(\prod_{i=1}^{k} U_{p_i,m_i} U^*_{p_i,n_i} \right) \langle \boldsymbol{m} | \rho^{(k)}_{\mathrm{ext}} | \boldsymbol{n} \rangle \,.\end{aligned} \tag{S6}$$

We consider an even number N of particles scattering in an N-mode interferometer made of $N/2$ beam splitters in parallel, as depicted in Fig. S1 for $N = 6$, such that

$$\begin{pmatrix} b_{2j-1,\alpha} \\ b_{2j,\alpha} \end{pmatrix} = \frac{1}{\sqrt{2}} \begin{pmatrix} 1 & 1 \\ 1 & -1 \end{pmatrix} \begin{pmatrix} a_{2j-1,\alpha} \\ a_{2j,\alpha} \end{pmatrix}, \tag{S7}$$

for $j = 1, \ldots N/2$. The index $\alpha \in \{H, V\}$ denotes the polarization state, which is assumed to be unaffected by the interferometer.

We want to evaluate the expectation value $\langle N_1 N_2 \ldots N_N \rangle$ in the following entangled states with one particle per input mode:

$$|\Psi_{\mathrm{Bell}}\rangle = \frac{a^\dagger_{1,H} + e^{i\varphi} a^\dagger_{1,V}}{\sqrt{2}} \left(\prod_{j=1}^{N/2-1} \frac{a^\dagger_{2j,H} a^\dagger_{2j+1,V} + e^{-i\chi_j} a^\dagger_{2j,V} a^\dagger_{2j+1,H}}{\sqrt{2}} \right) \frac{a^\dagger_{N,H} + e^{i\theta} a^\dagger_{N,V}}{\sqrt{2}} |0\rangle \tag{S8}$$

$$|\Psi_{\mathrm{GHZ}}\rangle = \frac{1}{\sqrt{2}} \left(\prod_{j=1}^{N/2} a^\dagger_{2j,H} + e^{-i\chi} \prod_{j=1}^{N/2} a^\dagger_{2j,V} \right) \prod_{j=1}^{N/2} \frac{a^\dagger_{2j-1,H} + e^{i\varphi_j} a^\dagger_{2j-1,V}}{\sqrt{2}} |0\rangle \,, \tag{S9}$$

corresponding to the schemes in Fig. S1(a) and Fig. S1(b), respectively, considering, for example, $N = 6$ particles.

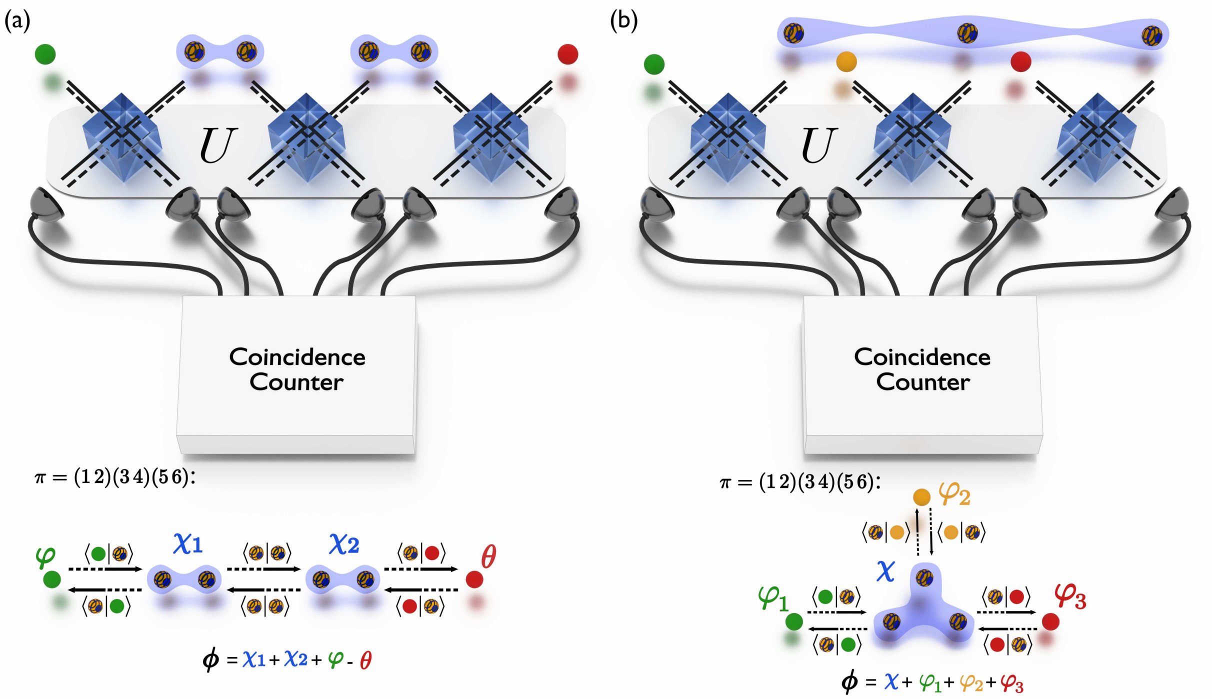

Figure S1: **Entanglement-induced collective interference of N particles. (a)** The use of $(N-2)/2$ (Bell) entangled particle pairs (blue envelopes) and $N/2$ separate beam splitters arranged in parallel between two particles with separable states (green and red particles), as depicted here for $N = 6$, results in a genuine NP interference process depending on the NP collective phase term ϕ associated with the permutation $\pi = (1\,2)\ldots(N-1\,N)$ (see bottom of the panel). **(b)** Analogously to **(a)**, the employment of an $N/2$-particle (GHZ) entangled state (blue envelope) and $N/2$ particles in separable states at the input ports of $N/2$ separate beam splitters, as shown here for $N = 6$, results in a genuine NP interference process depending on the NP collective phase term ϕ associated with the permutation $\pi = (1\,2)\ldots(N-1\,N)$ (see bottom of the panel).

We present two distinct derivations leading to the same result. The first approach employs a HOM-like scenario applied to the $N/2$ pairs of particles at the input ports of the $N/2$ beam-splitters. Conversely, the second derivation directly investigates the reduced external state $\rho_{\text{ext}}^{(N)}$.

Derivation based on HOM

Let us decompose the product $b_{2j-1,\alpha}b_{2j,\beta}$ into a symmetric ("triplet") and an antisymmetric ("singlet") component, which each transforms in an elementary fashion under the beam splitter unitary:

$$b_{2j-1,\alpha}b_{2j,\beta} = \frac{T_{j,\alpha,\beta} + S_{j,\alpha,\beta}}{\sqrt{2}}, \tag{S10}$$

$$T_{j,\alpha,\beta} = \frac{b_{2j-1,\alpha}b_{2j,\beta} + b_{2j,\alpha}b_{2j-1,\beta}}{\sqrt{2}} = \frac{a_{2j-1,\alpha}a_{2j-1,\beta} - a_{2j,\alpha}a_{2j,\beta}}{\sqrt{2}}, \tag{S11}$$

$$S_{j,\alpha,\beta} = \frac{b_{2j-1,\alpha}b_{2j,\beta} - b_{2j,\alpha}b_{2j-1,\beta}}{\sqrt{2}} = \frac{a_{2j-1,\alpha}a_{2j,\beta} - a_{2j,\alpha}a_{2j-1,\beta}}{\sqrt{2}}. \tag{S12}$$

We therefore have

$$N_{2j-1}N_{2j} = \sum_{\alpha,\beta} b^\dagger_{2j-1,\alpha}b^\dagger_{2j,\beta}b_{2j-1,\alpha}b_{2j,\beta} \tag{S13}$$

$$= \frac{1}{2}\sum_{\alpha,\beta}(T^\dagger_{j,\alpha,\beta} + S^\dagger_{j,\alpha,\beta})(T_{j,\alpha,\beta} + S_{j,\alpha,\beta}) \tag{S14}$$

$$= S^\dagger_j S_j + R_j$$

(S15)

where

$$S_j = S_{j,H,V} = -S_{j,V,H} = \frac{a_{2j-1,H}a_{2j,V} - a_{2j-1,V}a_{2j,H}}{\sqrt{2}}, \tag{S16}$$

while R_j collects terms where $T_{j,\alpha,\beta}$ or $T^\dagger_{j,\alpha,\beta}$ appears, which do not contribute to the expectation value in states with one particle per mode. The operator $S^\dagger_j$ creates a $|\psi^-\rangle$ Bell state in modes $2j-1$ and $2j$, which is invariant under the beam splitter transformation. For an input state $|\Psi\rangle$

with one particle per mode, the expectation value $\langle\Psi|N_1N_2\dots N_N|\Psi\rangle$ can thus be written as the overlap of the input state with a product of Bell states:

$$\langle\Psi|N_1N_2\dots N_N|\Psi\rangle = \left\|\prod_{j=1}^{N/2} S_j\,|\Psi\rangle\right\|^2 = |\langle\Psi|\Psi_{\mathrm{S}}\rangle|^2 \tag{S17}$$

with

$$|\Psi_{\mathrm{S}}\rangle = \prod_{j=1}^{N/2} S_j^\dagger\,|0\rangle = \prod_{j=1}^{N/2} \frac{a^\dagger_{2j-1,H}a^\dagger_{2j,V} - a^\dagger_{2j-1,V}a^\dagger_{2j,H}}{\sqrt{2}}\,|0\rangle\,. \tag{S18}$$

When computing the overlap in Eq. (S17), the only contributing terms are those where the polarization alternates between even and odd sites, i.e. those proportional to

$$|HV\dots\rangle = \prod_{j=1}^{N/2} a^\dagger_{2j-1,H}a^\dagger_{2j,V}\,|0\rangle \tag{S19}$$

and

$$|VH\dots\rangle = \prod_{j=1}^{N/2} a^\dagger_{2j-1,V}a^\dagger_{2j,H}\,|0\rangle\,. \tag{S20}$$

We write

$$|\Psi_{\mathrm{Bell}}\rangle = \frac{1}{\sqrt{2}^{N/2+1}}\left(e^{i\theta - i\sum_{j=1}^{N/2-1}\chi_j}\,|HV\dots\rangle + e^{i\varphi}\,|VH\dots\rangle\right) + |R_{\mathrm{Bell}}\rangle\,, \tag{S21}$$

$$|\Psi_{\mathrm{GHZ}}\rangle = \frac{1}{\sqrt{2}^{N/2+1}}\left(e^{-i\chi}\,|HV\dots\rangle + e^{+i\sum_{j=1}^{N/2}\varphi_j}\,|VH\dots\rangle\right) + |R_{\mathrm{GHZ}}\rangle\,, \tag{S22}$$

$$|\Psi_{\mathrm{S}}\rangle = \frac{1}{\sqrt{2}^{N/2}}\left(|HV\dots\rangle + (-1)^{N/2}\,|VH\dots\rangle\right) + |R_{\mathrm{S}}\rangle\,, \tag{S23}$$

where $|R_{\mathrm{Bell}}\rangle$, $|R_{\mathrm{GHZ}}\rangle$ and $|R_{\mathrm{S}}\rangle$ collect terms where at least two neighbouring modes are associated with the same polarization state such that $\langle\Psi_{\mathrm{S}}|R_{\mathrm{Bell}}\rangle = \langle\Psi_{\mathrm{S}}|R_{\mathrm{GHZ}}\rangle = \langle R_{\mathrm{S}}|\Psi_{\mathrm{Bell}}\rangle = \langle R_{\mathrm{S}}|\Psi_{\mathrm{GHZ}}\rangle = 0$. Therefore, from Eq. (S17), we conclude that both expectation values for $|\Psi_{\mathrm{Bell}}\rangle$ and $|\Psi_{\mathrm{GHZ}}\rangle$ can be written as

$$\langle\Psi|N_1N_2\dots N_N|\Psi\rangle = \frac{1}{2^N}\left(1 + (-1)^{N/2}\cos\phi\right)\,, \tag{S24}$$

with the many particle-phases

$$\phi_{\text{Bell}} = \sum_{j=1}^{N/2-1} \chi_j + \varphi - \theta \quad \text{and} \quad \phi_{\text{GHZ}} = \sum_{j=1}^{N/2} \varphi_j + \chi. \tag{S25}$$

Derivation using reduced state

We write the particle-number correlation between output modes $2j-1$ and $2j$ as

$$\begin{aligned} N_{2j-1}N_{2j} &= \sum_{\alpha,\beta} b^\dagger_{2j-1,\alpha} b^\dagger_{2j,\beta} b_{2j-1,\alpha} b_{2j,\beta} \\ &= \frac{1}{2}\sum_{\alpha,\beta}\left(a^\dagger_{2j-1,\alpha}a^\dagger_{2j,\beta} - a^\dagger_{2j,\alpha}a^\dagger_{2j-1,\beta}\right) a_{2j-1,\alpha}a_{2j,\beta} + R_j \end{aligned} \tag{S26}$$

where R_j collects terms containing two creation and/or two annihilation operators associated with the same mode, whose expectation value in a state with one particle per mode vanishes. Note that terms associated with identical internal states ($\alpha = \beta$) cancel out. The product of all N number operators reads

$$N_1 N_2 \dots N_N = \sum_{\boldsymbol{\alpha}} \sum_{\pi \in G} \frac{\text{sign}(\pi)}{2^{N/2}} a^\dagger_{\pi(\boldsymbol{p}),\boldsymbol{\alpha}} a_{\boldsymbol{p},\boldsymbol{\alpha}} + R \tag{S27}$$

where $\boldsymbol{p} = (1, 2, \dots N)$, G is the subgroup of S_N generated by transpositions $(2j-1\ 2j)$, $\text{sign}(\pi)$ is the parity of the number of such transpositions in π and R again collects terms which do not contribute in states with one particle per mode. For such a state ρ, we thus have (compare with Eq. (S6))

$$\langle N_1 N_2 \dots N_N \rangle = N! \sum_{\pi \in G} \frac{\text{sign}(\pi)}{2^{N/2}} \langle \boldsymbol{p} | \rho^{(N)}_{\text{ext}} | \pi(\boldsymbol{p}) \rangle \, . \tag{S28}$$

We now consider the specific input states $\rho = |\Psi\rangle\langle\Psi|$, with $|\Psi\rangle$ from Eqs. (S8) and (S9), which we write in the fashion of Eqs. (S21) and (S22) as

$$|\Psi_{\text{Bell}}\rangle = \frac{1}{\sqrt{2}^{N/2+1}} \left(e^{i\theta - i\sum_{j=1}^{N/2-1}\chi_j} a^\dagger_{\boldsymbol{p},\boldsymbol{\alpha}_0} |0\rangle + e^{i\varphi} a^\dagger_{\pi_0(\boldsymbol{p}),\boldsymbol{\alpha}_0} |0\rangle \right) + |R_{\text{Bell}}\rangle \, , \tag{S29}$$

$$|\Psi_{\text{GHZ}}\rangle = \frac{1}{\sqrt{2}^{N/2+1}} \left(e^{-i\chi} a^\dagger_{\boldsymbol{p},\boldsymbol{\alpha}_0} |0\rangle + e^{-i\sum_{j=1}^{N/2}\varphi_j} a^\dagger_{\pi_0(\boldsymbol{p}),\boldsymbol{\alpha}_0} |0\rangle \right) + |R_{\text{GHZ}}\rangle \, , \tag{S30}$$

with the alternating list of internal states $\boldsymbol{\alpha}_0 = (H, V, H, V \dots)$, the permutation $\pi_0 = (1\,2), (3\,4), \dots, (N-1\;N) \in G$, such that $\mathrm{sign}(\pi_0) = (-1)^{N/2}$, and $|R_{\mathrm{Bell}}\rangle$, $|R_{\mathrm{GHZ}}\rangle$ collecting terms where the polarization is the same in at least one pair of modes $2j-1$ and $2j$, which vanish upon applying $N_{2j-1}N_{2j}$. The relevant matrix elements of the reduced external state $\rho_{\mathrm{ext}}^{(N)}$ are thus found to be

$$\langle \boldsymbol{p}|\rho_{\mathrm{ext}}^{(N)}|\pi(\boldsymbol{p})\rangle = \frac{1}{N!}\frac{1}{2^{N/2}}\begin{cases}1 & \text{if } \pi = \mathrm{id}\\ \cos\phi & \text{if } \pi = \pi_0\\ 0 & \text{for other } \pi \in G\end{cases} \tag{S31}$$

such that

$$\langle N_1 N_2 \dots N_N\rangle = \frac{1}{2^N}\left(1 + (-1)^{N/2}\cos\phi\right), \tag{S32}$$

with the many particle phases ϕ from Eq. (S25).

Supplementary Note 2: Correlation versus coincidence measurements

The operator associated with the measurement of *at least one* photon in output mode p is given by (disregarding internal degrees of freedom)

$$\begin{aligned} M_p &= \sum_{n=1}^{\infty}\frac{(-1)^{n+1}}{n!}(a_p^\dagger)^n a_p^n && \text{(S33)}\\ &= \sum_{n=1}^{\infty}\frac{(-1)^{n+1}}{n!}N_p(N_p-1)\dots(N_p-n+1). && \text{(S34)}\end{aligned}$$

Indeed, the number state $|m\rangle$ with m photons in mode p satisfies

$$\begin{aligned} (\mathbb{1} - M_p)\,|m\rangle &= \sum_{n=0}^{\infty}\frac{(-1)^n}{n!}(a_p^\dagger)^n a_p^n\,|m\rangle && \text{(S35)}\\ &= \sum_{n=0}^{m}\frac{m!}{n!(m-n)!}(-1)^n\,|m\rangle && \text{(S36)}\\ &= \delta_{m,0}\,|m\rangle\,, && \text{(S37)}\end{aligned}$$

where we have made use of the binomial formula. Therefore, k-fold coincidence rates are obtained as the expectation value of

$$M_{p_1}\dots M_{p_k}. \tag{S38}$$

The lowest-order term in the expansion of Eq. (S38) is the k-point correlator $N_{p_1}\dots N_{p_k}$, corresponding to taking terms with $n = 1$ in Eq. (S33). This leading term is corrected by higher-order observables, which are sensitive to interference from higher orders than k.

As an example, when considering a system of $N = 4$ particles and neglecting terms beyond the fourth order, the explicit expansion of Eq. (S38) reads

$$\begin{aligned}
M_{p_1} &= N_{p_1} - \frac{1}{2}N_{p_1}(N_{p_1}-1) + \frac{1}{6}N_{p_1}(N_{p_1}-1)(N_{p_1}-2) \\
&\quad - \frac{1}{24}\boldsymbol{N_{p_1}(N_{p_1}-1)(N_{p_1}-2)(N_{p_1}-3)} && \text{(S39)}\\
M_{p_1}M_{p_2} &= N_{p_1}N_{p_2} - \frac{1}{2}N_{p_1}(N_{p_1}-1)N_{p_2} - \frac{1}{2}N_{p_1}N_{p_2}(N_{p_2}-1) \\
&\quad + \frac{1}{4}\boldsymbol{N_{p_1}(N_{p_1}-1)N_{p_2}(N_{p_2}-1)} + \frac{1}{6}\boldsymbol{N_{p_1}(N_{p_1}-1)(N_{p_1}-2)N_{p_2}} \\
&\quad + \frac{1}{6}\boldsymbol{N_{p_1}N_{p_2}(N_{p_2}-1)(N_{p_2}-2)} && \text{(S40)}\\
M_{p_1}M_{p_2}M_{p_3} &= N_{p_1}N_{p_2}N_{p_3} - \frac{1}{2}\boldsymbol{N_{p_1}(N_{p_1}-1)N_{p_2}N_{p_3}} - \frac{1}{2}\boldsymbol{N_{p_1}N_{p_2}(N_{p_2}-1)N_{p_3}} \\
&\quad - \frac{1}{2}\boldsymbol{N_{p_1}N_{p_2}N_{p_3}(N_{p_3}-1)} && \text{(S41)}\\
M_{p_1}M_{p_2}M_{p_3}M_{p_4} &= \boldsymbol{N_{p_1}N_{p_2}N_{p_3}N_{p_4}}, && \text{(S42)}
\end{aligned}$$

where fourth-order terms are highlighted. The presence of fourth-order terms in Eqs. (S39)-(S41) results in a residual dependence of $k(< N)$-fold coincidences on the collective phase ϕ, where the magnitude of this influence increases as k approaches N. The presence of these higher-order contributions is illustrated in Fig. **S2**: numerical simulations indeed show that three-fold coincidences depend on ϕ, but with much weaker visibility than for the four-fold coincidence.

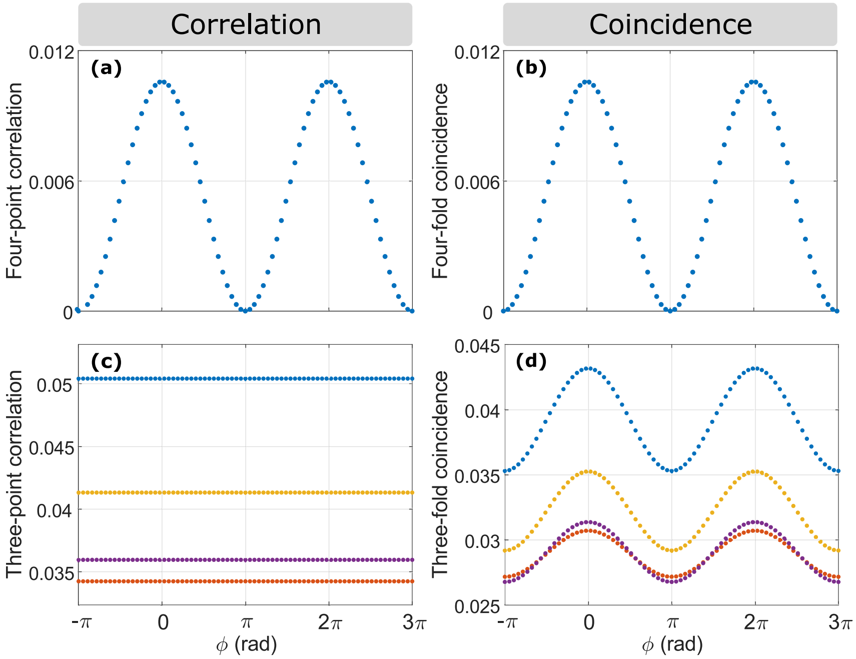

Figure S2: **Simulations of correlations and coincidence events.** Correlations show **(a)** a fully visible fringe pattern of the four-point correlator, whereas **(c)** the three-point correlators, which depend on the coherence of the reduced three-particle states, are all independent of the collective phase. We sampled input and output transmission efficiencies from a normal distribution with a mean value of 0.8 and a standard deviation of 0.1. Different total losses displace the curves among each other, with higher losses resulting in lower curves. Simulations of coincidence events show **(b)** four-fold coincidence with same behaviour as the four-point correlation (see Eq. (S42)), and **(d)** a signature of the collective 4P phase also in the three-fold coincidences, as these are not proper 3P measurements. The visibility of this signature strongly depends on the optical losses, with lower values as losses increase.

To grasp the dependence of three-fold coincidences on the collective phase ϕ, consider the three-fold events among, for example, the output channels A, C, and D in the lossless conditions where $\phi = \pi,\, 0$. The output mode-occupation lists (counting the number of particles in each output mode) contributing to such events are $\mathbf{s_1} = (2,\, 0,\, 1,\, 1)$ and $\mathbf{s_2} = (1,\, 1,\, 1,\, 1)$. When $\phi = \pi$, the absence of four-fold coincidences indicates a bunching behaviour of photons, resulting in the suppression of events corresponding to $\mathbf{s_2}$ and in a three-fold coincidence probability solely given by $\mathbf{s_1}$, i.e., $p(\mathrm{ACD}|\phi = \pi) = p(\mathbf{s_1}|\phi = \pi)$. For symmetry reasons, $\mathbf{s_1}$ must have the same probability as the output state $\mathbf{s_3} = (0,\, 2,\, 1,\, 1)$ (which does not produce a coincidence in A, C and D). When $\phi = 0$, all three states $\mathbf{s_1}$, $\mathbf{s_2}$, and $\mathbf{s_3}$ are allowed, of which $\mathbf{s_2}$ can be achieved via two distinct exchange processes at the beam splitter connecting modes A and B (double reflection and double transmission), while $\mathbf{s_1}$ and $\mathbf{s_3}$ can only be obtained via a single process each. Intuitively, this halves the probability of $\mathbf{s_1}$ with respect to the $\phi = \pi$-scenario, whereas the probability of $\mathbf{s_2}$ is double the probability of $\mathbf{s_1}$, that is, $p(\mathbf{s_2}|\phi = 0) = p(\mathbf{s_1}|\phi = \pi)$. Therefore, the three-fold coincidence probability becomes $p(\mathrm{ACD}|\phi = 0) = p(\mathbf{s_1}|\phi = 0) + p(\mathbf{s_2}|\phi = 0) = p(\mathbf{s_1}|\phi = \pi)(\frac{1}{2} + 1) = \frac{3}{2}p(\phi = \pi)$. When losses are considered, such as in Fig. S2, the factor $3/2$ is reduced because three-fold coincidences from $\mathbf{s_1}$ are less affected by losses with respect to $\mathbf{s_2}$ (one photon loss in channel A of $\mathbf{s_1}$ still result in a coincidence).

Simulations of coincidences with the addition of one detector multiplexing layer, as in Fig. (2), predict a further visibility reduction of a factor ~ 2.3 (influenced by the value of the total optical losses) in the three-fold coincidences fluctuations of Fig. S2(d).

Supplementary Note 3: State fidelity

We evaluated the state overlap, referred to as fidelity *(61)*, of the entangled state of the photons in modes 2 and 3 with respect to the Bell states $|\psi^+\rangle$, $|\psi^-\rangle$, corresponding to setting $\chi =$

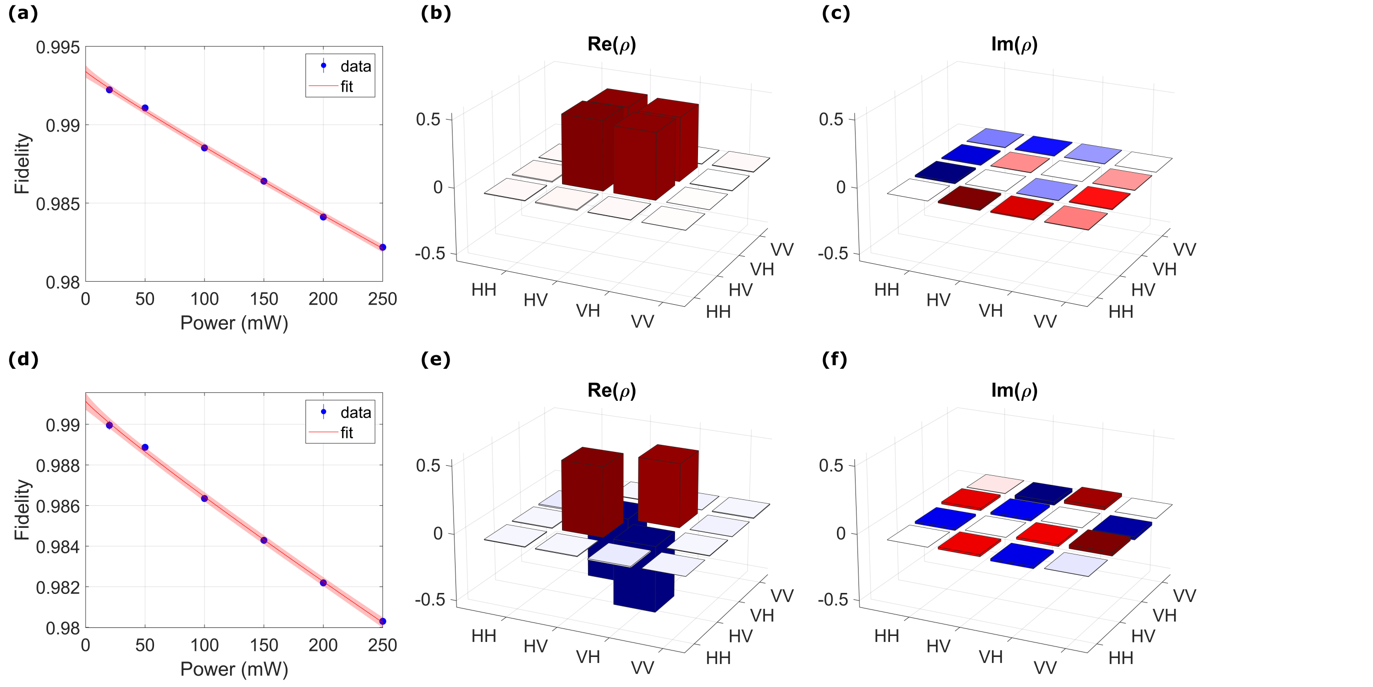

Figure S3: **Tomography of the entangled photon pair.** The panels in the upper and lower rows are the results for the input states $|\psi^+\rangle$ and $|\psi^-\rangle$ of the photons in modes 2 and 3, respectively. Panels **(a)** and **(d)** show the dependence of the states' fidelities on the laser pump power, whereas the other panels show the real (**(b)** and **(e)**) and imaginary (**(c)** and **(f)**) parts of the reconstructed density matrix ρ for a representative value of $150\,\mathrm{mW}$.

$0, \pi$ in Eq. (3), by performing a maximum likelihood state tomography *(62)*. To this end, the source outputs are connected to two polarisation-tomography stages where we measure single counts and coincidence events. As mentioned in the main text, the phase χ is adjusted via fibre polarisation controllers. We performed six state tomographies for the average pump power range $20\,\mathrm{mW}$–$250\,\mathrm{mW}$, which includes the power used to perform the experiment $\simeq 174\,\mathrm{mW}$, and we evaluated the fidelity of the resulting density matrices with the corresponding states $|\psi^+\rangle$, $|\psi^-\rangle$.

The results of these measurements are shown in Fig. **S3**. From the fit of the data, we find a fidelity at $174\,\mathrm{mW}$ of 98.53(2)% and 98.33(2)%, respectively, for $|\psi^+\rangle$ and $|\psi^-\rangle$.

Supplementary Note 4: Raw data

We present here the raw data of the background-subtracted measurement in Fig. (3). As described in the main text, we performed φ-phase scans at fixed phases $\chi = 0, \pi$, and $\theta = 0$ in Eqs. (3)-(4). We first show the results of single-source measurements (when only one source is pumped by the laser) and, subsequently, the results of the experiment when background subtraction is not employed.

Single-source measurements provide a useful comparison for the expected fluctuations of single counts and coincidence events in the absence of interference of photons from different sources. In addition, since photons from one source are coupled to separate beam splitters (cf. Fig. (2)), also no interference effect between photons from the same source can take place, and fluctuations can be purely associated with other contributions, such as residual polarisation dependence of the system.

We report in Fig. **S4** the measurements of single counts and coincidence events (together with the simulations discussed in Supplementary Note 5) for the two separate sources when $\chi = \pi$, but similar results are obtained for $\chi = 0$. Moreover, all four-fold background coinci-

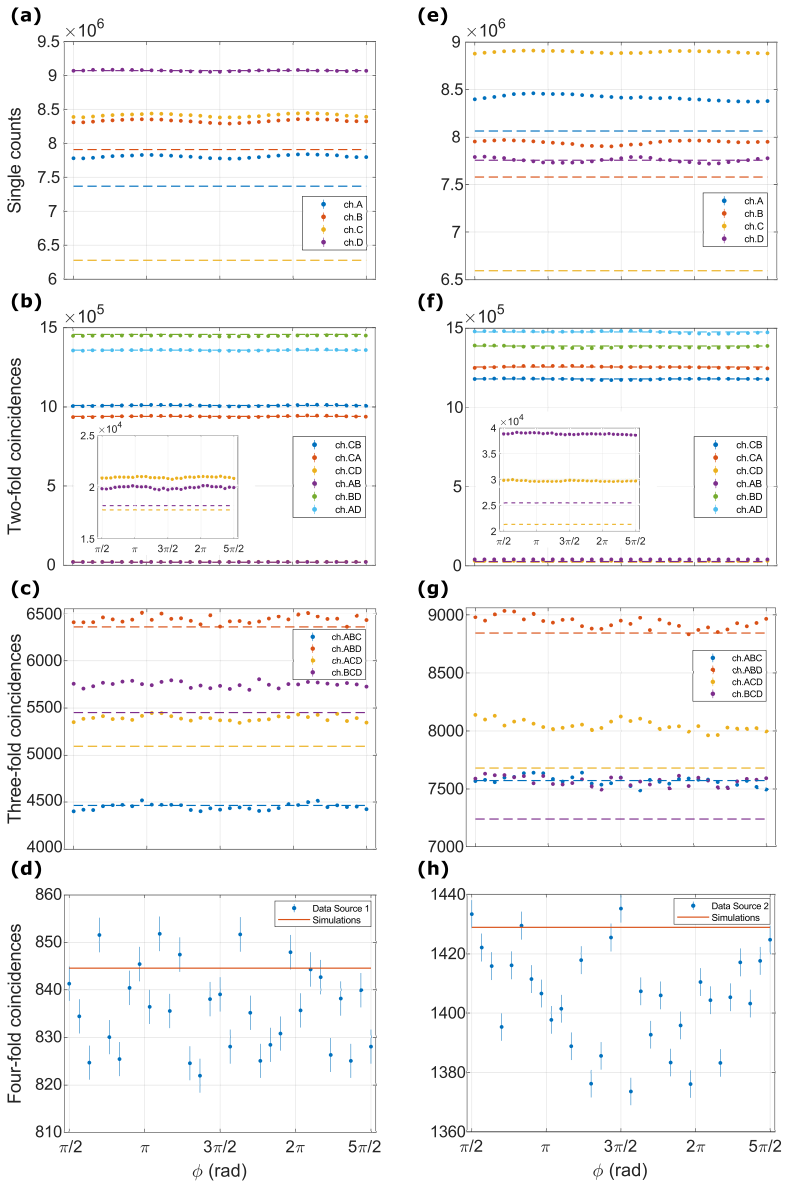

Figure S4: **Measurements and simulations of the single-source measurements for $\chi = \pi$ in Eq. (3).** Panels **(a)**-**(d)** report single counts and coincidence events measured when blocking the source of separable photons (Source 1). The panels also show the corresponding results achieved via simulations based on the optimisation algorithm discussed in Supplementary Note 5. Panels **(e)**-**(h)** report the same results obtained when blocking the source of entangled photons (Source 2). In all graphs, the integration time for each point is $60\,\mathrm{s}$.

dence measurements (for both $\chi = 0,\ \pi$) can be found in Figs. (3)(a) and (3)(b). Single counts in Figs. **S4**(a) and **S4**(e) exhibit relative fluctuations of the order of $\simeq 0.2\%$. Similarly to single counts, two-, three-, and four-fold coincidence events have only marginal fluctuations, respectively of the order of $\simeq 0.3\%$, $\simeq 0.5\%$ and $\simeq 1.2\%$. The increasing value in the fluctuations of coincidence events is mainly given by the smaller photon counting statistics of these cases.

Fig. **S5** shows the data collected when both sources are employed, before subtraction of the background, for both settings of the phase $\chi = 0,\ \pi$ (with $\theta = 0$).

The single counts in Figs. **S5**(a) and **S5**(e) show only small changes as we vary the collective phase ϕ, with relative fluctuations of $\simeq 0.1 - 0.2\%$. This value is consistent with the corresponding fluctuations of single counts measured when using only one source at a time, where the smaller sample size produces slightly higher values.

Two-fold coincidences in Figs. **S5**(b) and **S5**(f) have relative fluctuations of the order of $\simeq 1.7\%$ and $\simeq 2.4\%$, respectively. These values are larger than the corresponding ones measured with the single sources ($\simeq 0.3\%$). Nevertheless, they are connected to interference effects between the input states produced by the two sources *(18)* that cannot be attributed to a dependence on the collective phase. In particular, these fluctuations do not show the expected cosinusoidal dependence on the collective phase, but rather exhibit a complex interference pattern that arises as a consequence of the relative phase between the dominant single-pair emission contributions of the two sources. In fact, two-fold coincidences of channels associated with the same beam splitter — chs. A-B or chs. C-D in Fig. (2) —, which are not influenced by these contributions, exhibit significantly weaker fluctuations ($\simeq 0.3 - 0.4\%$), as shown in the insets of Figs. **S5**(b) and **S5**(f), despite their reduced photon counting statistic. Specifically, the partial connectivity of the interferometer does not allow single-pair emissions from one source to contribute to two-fold coincidences of chs. A-B or chs. C-D, which necessitate two-pair emissions. This requirement prevents two-fold coincidences between these channels from only two

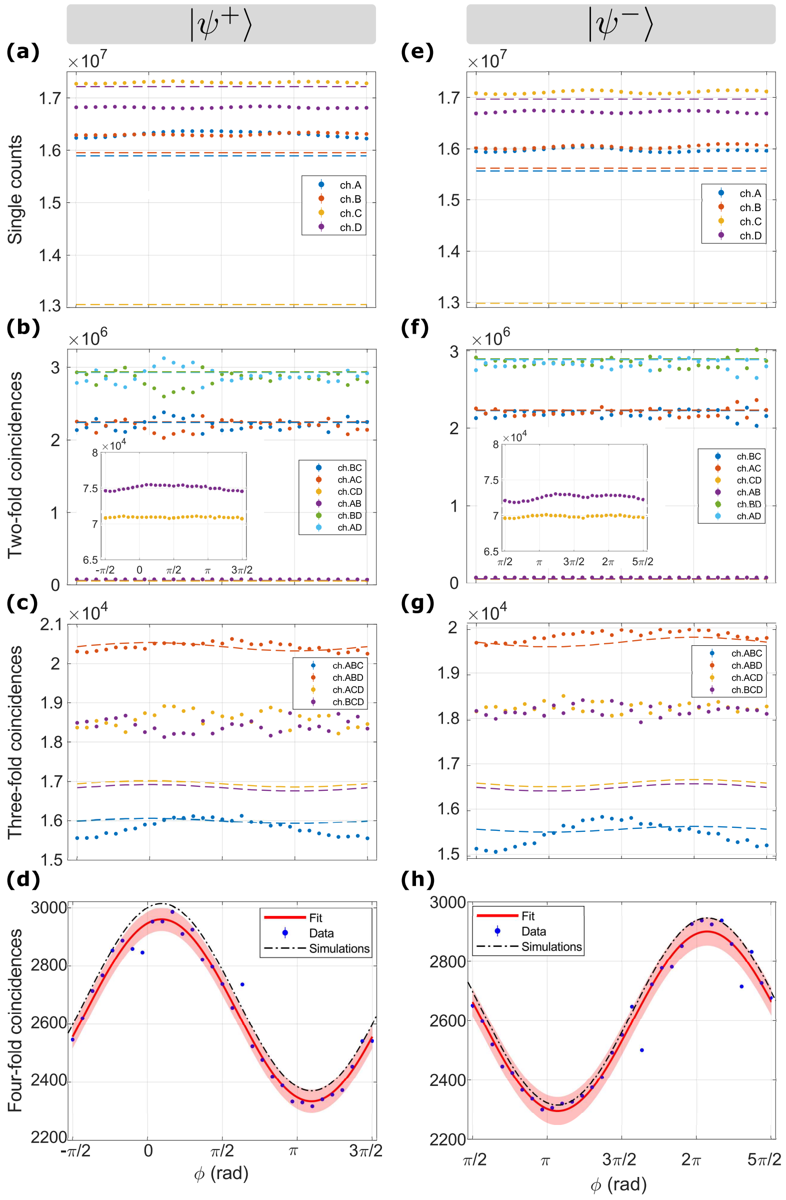

Figure S5: **Measurements of single and coincidence counts for two settings of the entangled state phase** $\chi = 0,\, \pi$ **in Eq. (3)** ($|\psi^+\rangle$ and $|\psi^-\rangle$ in panels (a)-(d) and (e)-(h), respectively). **(a)(e)** Single counts. **(b)(f)** Two-fold coincidences. **(c)(g)** Three-fold coincidences. **(d)(h)** Four-fold coincidences data (blue dots) fitted with a cosine function (red curve). The red-shaded region shows the fit prediction interval at a confidence level of one standard deviation. The graphs include the results of the multiphoton interference simulations (black dashed curve). The visibility of the fitting cosine is $11.6(5)\%$ and $11.8(4)\%$ for insets (d) and (h), whereas simulations predict a visibility of $12.0(1)\%$ and $11.9(1)\%$, respectively. In all graphs, the integration time for each point is $60\,\mathrm{s}$.

particles in the system and ensures contributions from a wider spectrum of interfering terms, originating from all exchange processes of at least four particles at the two beam splitters, thus reducing the influence of relative phase terms and, consequently, possible fluctuations. Therefore, the coincidences between chs. A-B and chs. C-D are a good indicator for the independence of two-fold coincidences from the collective 4P phase. Of these, chs. C-D do not exhibit a clear dependence on ϕ, as shown in the two insets, whereas chs. A-B have slightly larger fluctuations with a specific pattern displaying a maximum in the centre of the ϕ range (corresponding to half the rotation range of the half-wave plate in input mode 1). In contrast, a pure dependence on the collective phase ϕ would lead to a π-shift, i.e., an inversion of the pattern between the two curves for $|\psi^+\rangle$ and $|\psi^-\rangle$, which is clearly not the case. Therefore, we conclude that this fluctuation pattern is dominated by artefacts of the wave-plate rotation rather than the collective phase.

Three-fold coincidences in Figs. S5(c) and S5(g) have residual interference effects similar to two-fold coincidences that appear as anti-correlated patterns of coincidences comprising only one of the output channels A and B. The influence of such interference effects on the three-fold coincidences is already significantly weaker with respect to the two-fold case, with fluctuations of the order of $\simeq 0.8\%$. Conversely, the largest fluctuations are here connected to three-fold coincidences among chs. A-B-C, which, however, show a pattern that, in both cases, can be correlated to the two-fold coincidence data of chs. A-B (Pearson correlation coefficients $\simeq 0.91$). Consequently, fluctuations are once more associated with unwanted artefacts and residual interference effects, instead of a dependence on the collective phase.

While two-fold and three-fold coincidences do not exhibit any obvious dependence on the collective phase ϕ, the four-fold coincidences reported in Figs. S5(d) and S5(h) show a clear oscillation. In particular, the four-fold coincidences are in excellent agreement with the cosine behaviour predicted by Eq. (5), showing relatively high visibility compared to the previous

cases of $11.6(5)\%$ and $11.8(4)\%$, which excludes the possibility that the observed four-fold oscillations are an artefact of the interference effects mentioned above for two- and three-fold coincidences. Moreover, as also shown in the main text, the experimental data exhibit a good agreement with numerical simulations of the experiment (further details in Supplementary Note 5).

Supplementary Note 5: Simulations

To further support the experimental results, we performed simulations of the experiment based on the specific experimental scheme and the photon sources used to witness the entanglement-induced collective interference.

For this purpose, we performed a characterisation of the two SPDC sources and developed a model of the unitary transformation realised in Fig. (2)(b). Source characterisation is required to calculate the emission rates of single-pair emissions and higher-order emissions from the SPDC processes, depending on the laser pump power and pulse repetition rate. The unitary transformation model, instead, allows us to evaluate the probability of all possible output events for each input state corresponding to combinations of different emission orders from the two sources. The reconstruction of the unitary transformation is achieved via an optimisation algorithm based on measurements of single count events — the detection events measured by each detector independently of the measurements of all other detectors — and all k-fold coincidence rates, with $k = 2, 3, 4$, produced by the individual sources. As a result, we can calculate the total single counts and coincidence rates by using the emission rates of each input state and the relative output probabilities.

The simulations take into account a few fundamental approximations. First, the two sources are considered independent of each other. This is strictly true only when we use single counts and coincidence rates from single source emissions, i.e., the data exploited for the reconstruction

of the unitary transformation. In fact, as already mentioned in Supplementary Note 4, the presence of phase terms among the input states of the combined two-source SPDC process can give rise to interference in the coincidence rates. Second, it is assumed that the down-conversion process of the pump photons results in spectrally pure down-converted photon pairs, corresponding to fully separable output photon states — the production of entangled pairs is only associated with the geometry of the Sagnac interferometers *(46)*. This approximation is well justified due to the high spectral purity ($\geq 98\%$) of the SPDC sources used in the experiment, as extensively studied in *(45)*. Partial distinguishability of the input particles (see Eqs. (3)-(4)) is obtained in terms of polarisation states, which, following the formalism developed in *(63)*, are considered as internal degrees of freedom of the input state. While this is computationally efficient, the unitary transformation is unable to act on the photons' transformation depending on their polarisation state, thus excluding any modelling of residual polarisation dependence of losses and beam splitters' splitting ratios. However, this limitation does not significantly influence the results to a first approximation.

Photon sources

The photon sources in Fig. (2)(a) are modeled with two independent SPDC processes, where we expressed the resulting photon states in terms of two-mode squeezed vacuum states in the photon number basis n:

$$|\zeta\rangle = \sqrt{1-\eta^2}\sum_{n=0}^{\infty}\eta^n\,|n,n\rangle_{s,i}\,, \tag{S43}$$

with s and i indicating the signal and idler modes, and η the squeezing parameter. This photon state represents a good approximation for calculating the emission rates even for the bidirectionally pumped crystal in Fig. (2)(a) (used to produce entangled pairs). The emission probability of a specific photon number state $|n,n\rangle_{s,i}$ per laser pulse is associated with the squeezing parameter by $P(n) = (1-\eta^2)\eta^{2n}$. Moreover, the squeezing parameter can be expressed as $\eta = \sqrt{\tau p}$, in

terms of the pump power p and a constant τ that quantifies the nonlinear interaction within the aKTP crystals *(45, 64)*. Similarly to *(64)*, we calculated τ for both sources by measuring single counts and coincidence rates and knowing the repetition rate of the laser pulses f (80 MHz). Specifically, by considering an input optical power of 174 mW and 168 mW for Source 1 and Source 2, respectively, we obtained squeezing parameters of $\eta_{S_1} = 0.102$ and $\eta_{S_2} = 0.094$.

The evaluation of η allows us to determine the single photon pair rates and the rates of higher-order pair productions depending on the pump power. To show this, by using the polarisation basis, the overall state of the down-converted photons collected at the output of the Sagnac interferometers can be written as:

$$|\psi\rangle = |\zeta_{S_1}\rangle \otimes |\zeta_{S_2}\rangle = \\ \Big(\sqrt{1-\eta_{S_1}^2}\sum_{n=0}^{\infty}\eta_{S_1}^n\big[\frac{1}{\sqrt{2}}(|H,V\rangle_{2,3} + e^{-i\chi}|V,H\rangle_{2,3})\big]^{\otimes n}\Big) \otimes \Big(\sqrt{1-\eta_{S_2}^2}\sum_{m=0}^{\infty}\eta_{S_2}^m |H,V\rangle_{1,4}^{\otimes m}\Big), \tag{S44}$$

where S_1 and S_2 refer to the source of entangled photon states (Source 1) and of separable photon states (Source 2), the indices $1,\ 2,\ 3,\ 4$ correspond to chs. 1-4 before the beam splitters in Fig. (2)(b), and χ is the usual phase of the entangled state as defined in Eq. (3). Starting from Eq. (S44), we can calculate the emission rates of each mode-occupation list $\mathbf{R} = (r_1, r_2, r_3, r_4)$ corresponding to the occupation number of each unitary input channel $1-4$ *(63)*. Given the nature of the SPDC process (two-photon emissions) and the arrangement of the channels in Fig. (2), we have $r_1 = r_4 := r_{S_2}$ and $r_2 = r_3 := r_{S_1}$, where r_{S_1} and r_{S_2} are defined as the number of pair emissions of each source. Therefore, each mode-occupation list $\mathbf{R}$ can be associated with an input state similar to Eq. (3):

$$|\xi\rangle = \left[\frac{1}{\sqrt{2}}(a^\dagger_{2,H}a^\dagger_{3,V} + e^{-i\chi}a^\dagger_{2,V}a^\dagger_{3,H})\right]^{r_{S_1}} (a^\dagger_{1,\mathrm{S}}(\varphi)a^\dagger_{4,\mathrm{S}}(\theta))^{r_{S_2}}|0\rangle\,. \tag{S45}$$

The state $|\xi\rangle$ is related to the contribution of a specific pair of values (m, n) in Eq. (S44), from which one can evaluate the emission rate by knowing η_{S_1}, η_{S_2}, and f.

For example, the input state in Eq. (3) is associated with $r_{S_1} = r_{S_2} = 1$, $\mathbf{R} = (1,1,1,1)$, i.e., a single firing of both sources. The contribution to this state is related to the $m = n = 1$ terms in Eq. (S44), whose emission rate is given by $r_{1,1} = fP(\mathbf{R}_{1,1}) = f(1-\eta^2_{S_1})\eta^2_{S_1}(1-\eta^2_{S_2})\eta^2_{S_2}$. In a similar fashion, one can calculate the rates of all mode-occupation lists resulting from all possible combinations of sources' emissions. We considered here contributions of up to three photon-pair productions, which can arise from both single-source emissions ($r_{S_1} = 3,\ r_{S_2} = 0$, or the opposite) or emissions from combinations of both sources ($r_{S_1} = 2,\ r_{S_2} = 1$, or the opposite).

Unitary reconstruction

Each mode-occupation list contributes differently to the total single counts and coincidence rates. In fact, each mode-occupation list has a different emission rate and probability of giving rise to specific output events after propagation through the interference setup. The calculation of these probabilities is based on the formalism developed in *(63)*, which, given a certain unitary transformation, provides the input-output probabilities for each input mode-occupation list $\mathbf{R}$.

We reconstructed the unitary transformation associated with Fig. (2)(b), given by the two independent beam splitters, to include the possibility of both optical losses before and after the two beam splitters, and unbalanced splitting ratios of the beam splitters themselves. Losses are modelled by extending the four-mode unitary with additional unbalanced beam splitters and external modes. We consider independent transmission factors η_j for each of the eight input and output modes of the beam splitters.

The multiplexed eight-detector scheme (inset of Fig. (2)(b)) is modelled via additional balanced beam splitters, randomly distributing the arriving photons on the two detectors of each output channel A-D. This is a valid simplification, as in the experiment the polarisation projection is only used to fix the photon polarisation on individual detectors. All losses of this second

transformation stage and the detector efficiencies are incorporated into the output channel losses of the main beam splitter unitary.

As a result, ten parameters are required to reconstruct the extended unitary: eight transmission coefficients η_j to model the input and output losses and the two splitting ratios of the main beam splitters. We employed an optimisation algorithm for these ten parameters, which aims to match the average values of the simulated and measured single counts and coincidence rates. More precisely, we carried out this parameter search by simultaneously emulating both single-source measurements performed by blocking one source at a time — thus matching eight single count rates, twelve two-fold coincidence rates, eight three-fold coincidence rates, and two four-fold coincidence rates. This procedure is used for both the two measurements with $\chi = 0, \pi$ to allow for marginal deviations in alignment between the two experimental conditions.

The panels in Fig. **S4** show the comparison between measurements and simulations for the case $\chi = \pi$, and similar results are obtained for $\chi = 0$. Consistently, the transmission coefficients of the main beam splitters do not differ between the two optimisations to all reasonable orders of approximation, and variations of input and output losses are of the order of $\pm 0.8\%$. Moreover, for the specific beam splitters used, we found deviations from a balanced 50:50 splitting ratio that are within the $\pm 3\%$ tolerance interval given by the producer *(65)*. The panels associated with the coincidence rates in Fig. **S4** show good agreement with the measurements, with relative deviations $< 5\%$. This is only partially true for single count rates, where channel C has a relative deviation of $\simeq 25\%$. Presumably, this is an effect of correlated loss affecting channel C, e.g. due to small errors in setting the FPGA unit's logic delays, which is hardly noticeable beforehand without applying this optimisation routine. This defect is modelled by the algorithm with an effective higher loss of channel C.

Power drifts

As mentioned in the main text, drifts in laser power between the main measurements in Fig. **S5** and the background measurements in Fig. **S4** lead to errors in the background subtraction.

Therefore, we performed another optimisation routine based on the single counts and all k-fold coincidence rates of the main measurements (again one optimisation for each phase $\chi = 0,\ \pi$ in Eq. (3)), but this time only allowing for a variation of the input power affecting both photon sources in the same manner. According to this procedure, the power is approximately unchanged for the measurement of $\chi = \pi$, demonstrating that no significant power fluctuation influences Fig. (3)(d), while a relative power drift of $\simeq 2.3\%$ affects the measurement of $\chi = 0$, thus resulting in the considerable visibility reduction obtained in Fig. (3)(c).

REFERENCES AND NOTES

1. R. Horodecki, P. Horodecki, M. Horodecki, K. Horodecki, Quantum entanglement. *Rev. Mod. Phys.* **81**, 865–942 (2009).

2. J. S. Bell, On the Einstein Podolsky Rosen paradox. *Phys. Phys. Fiz.* **1**, 195–200 (1964).

3. B. Hensen, H. Bernien, A. E. Dréau, A. Reiserer, N. Kalb, M. S. Blok, J. Ruitenberg, R. F. L. Vermeulen, R. N. Schouten, C. Abellán, W. Amaya, V. Pruneri, M. W. Mitchell, M. Markham, D. J. Twitchen, D. Elkouss, S. Wehner, T. H. Taminiau, R. Hanson, Loophole-free Bell inequality violation using electron spins separated by 1.3 kilometres. *Nature* **526**, 682–686 (2015).

4. H.-J. Briegel, W. Dür, J. I. Cirac, P. Zoller, Quantum repeaters: The role of imperfect local operations in quantum communication. *Phys. Rev. Lett.* **81**, 5932–5935 (1998).

5. Y.-A. Chen, S. Chen, Z.-S. Yuan, B. Zhao, C.-S. Chuu, J. Schmiedmayer, J.-W. Pan, Memory-built-in quantum teleportation with photonic and atomic qubits. *Nat. Phys.* **4**, 103–107 (2008).

6. D. P. Nadlinger, P. Drmota, B. C. Nichol, G. Araneda, D. Main, R. Srinivas, D. M. Lucas, C. J. Ballance, K. Ivanov, E. Y.-Z. Tan, P. Sekatski, R. L. Urbanke, R. Renner, N. Sangouard, J.-D. Bancal, Experimental quantum key distribution certified by Bell's theorem. *Nature* **607**, 682–686 (2022).

7. E. Knill, R. Laflamme, G. J. Milburn, A scheme for efficient quantum computation with linear optics. *Nature* **409**, 46–52 (2001).

8. R. Raussendorf, D. E. Browne, H. J. Briegel, Measurement-based quantum computation on cluster states. *Phys. Rev. A* **68**, 022312 (2003).

9. S. Bartolucci, P. Birchall, H. Bombín, H. Cable, C. Dawson, M. Gimeno-Segovia, E. Johnston, K. Kieling, N. Nickerson, M. Pant, F. Pastawski, T. Rudolph, C. Sparrow, Fusion-based quantum computation. *Nat. Commun.* **14**, 912 (2023).

10. L. Sansoni, F. Sciarrino, G. Vallone, P. Mataloni, A. Crespi, R. Ramponi, R. Osellame, Two-particle bosonic-fermionic quantum walk via integrated photonics. *Phys. Rev. Lett.* **108**, 010502 (2012).

11. J. C. F. Matthews, K. Poulios, J. D. A. Meinecke, A. Politi, A. Peruzzo, N. Ismail, K. Wörhoff, M. G. Thompson, J. L. O'Brien, Observing fermionic statistics with photons in arbitrary processes. *Sci. Rep.* **3**, 1539 (2013).

12. C. K. Hong, Z. Y. Ou, L. Mandel, Measurement of subpicosecond time intervals between two photons by interference. *Phys. Rev. Lett.* **59**, 2044–2046 (1987).

13. F. Bouchard, A. Sit, Y. Zhang, R. Fickler, F. M. Miatto, Y. Yao, F. Sciarrino, E. Karimi, Two-photon interference: The Hong-Ou-Mandel effect. *Rep. Prog. Phys.* **84**, 012402 (2020).

14. M. C. Tichy, Interference of identical particles from entanglement to boson-sampling. *J. Phys. B: At. Mol. Opt. Phys.* **47**, 103001 (2014).

15. C. Dittel, G. Dufour, G. Weihs, A. Buchleitner, Wave-particle duality of many-body quantum states. *Phys. Rev. X* **11**, 031041 (2021).

16. Y. L. Lim, A. Beige, Generalized Hong-Ou-Mandel experiments with bosons and fermions. *New J. Phys.* **7**, 155 (2005).

17. M. C. Tichy, M. Tiersch, F. D. Melo, F. Mintert, A. Buchleitner, Zero-transmission law for multiport beam splitters. *Phys. Rev. Lett.* **104**, 220405 (2010).

18. M. C. Tichy, H.-T. Lim, Y.-S. Ra, F. Mintert, Y.-H. Kim, A. Buchleitner, Four-photon indistinguishability transition. *Phys. Rev. A* **83**, 062111 (2011).

19. M.-O. Pleinert, A. Rueda, E. Lutz, J. von Zanthier, Testing higher-order quantum interference with many-particle states. *Phys. Rev. Lett.* **126**, 190401 (2021).

20. F. Flamini, N. Spagnolo, F. Sciarrino, Photonic quantum information processing: A review. *Rep. Prog. Phys.* **82**, 016001 (2018).

21. L. S. Madsen, F. Laudenbach, M. F. Askarani, F. Rortais, T. Vincent, J. F. F. Bulmer, F. M. Miatto, L. Neuhaus, L. G. Helt, M. J. Collins, A. E. Lita, T. Gerrits, S. W. Nam, V. D. Vaidya, M. Menotti, I.

Dhand, Z. Vernon, N. Quesada, J. Lavoie, Quantum computational advantage with a programmable photonic processor. *Nature* **606**, 75–81 (2022).

22. M. Tichy, F. de Melo, M. Kuś, F. Mintert, A. Buchleitner, Entanglement of identical particles and the detection process. *Fortschritte der Physik* **61**, 225–237 (2013).

23. Y.-S. Ra, M. C. Tichy, H.-T. Lim, O. Kwon, F. Mintert, A. Buchleitner, Y.-H. Kim, Nonmonotonic quantum-to-classical transition in multiparticle interference. *Proc. Natl. Acad. Sci. U.S.A.* **110**, 1227–1231 (2013).

24. M. Walschaers, J. Kuipers, J.-D. Urbina, K. Mayer, M. C. Tichy, K. Richter, A. Buchleitner, Statistical benchmark for BosonSampling. *New J. Phys.* **18**, 032001 (2016).

25. T. Giordani, F. Flamini, M. Pompili, N. Viggianiello, N. Spagnolo, A. Crespi, R. Osellame, N. Wiebe, M. Walschaers, A. Buchleitner, F. Sciarrino, Experimental statistical signature of many-body quantum interference. *Nat. Photonics* **12**, 173–178 (2018).

26. M. Pont, R. Albiero, S. E. Thomas, N. Spagnolo, F. Ceccarelli, G. Corrielli, A. Brieussel, N. Somaschi, H. Huet, A. Harouri, A. Lemaître, I. Sagnes, N. Belabas, F. Sciarrino, R. Osellame, P. Senellart, A. Crespi, Quantifying *n*-Photon Indistinguishability with a Cyclic Integrated Interferometer. *Phys. Rev. X* **12**, 031033 (2022).

27. E. Brunner, A. Buchleitner, G. Dufour, Many-body coherence and entanglement probed by randomized correlation measurements. *Phys. Rev. Res.* **4**, 043101 (2022).

28. B. Seron, L. Novo, N. J. Cerf, Boson bunching is not maximized by indistinguishable particles. *Nat. Photonics* **17**, 702–709 (2023).

29. A. Crespi, Suppression laws for multiparticle interference in Sylvester interferometers. *Phys. Rev. A* **91**, 013811 (2015).

30. C. Dittel, R. Keil, G. Weihs, Many-body quantum interference on hypercubes. *Quantum Sci. Technol.* **2**, 015003 (2017).

31. C. Dittel, G. Dufour, M. Walschaers, G. Weihs, A. Buchleitner, R. Keil, Totally destructive many-particle interference. *Phys. Rev. Lett.* **120**, 240404 (2018).

32. J. Münzberg, C. Dittel, M. Lebugle, A. Buchleitner, A. Szameit, G. Weihs, R. Keil, Symmetry allows for distinguishability in totally destructive many-particle interference. *PRX Quantum* **2**, 020326 (2021).

33. W. S. Warren, S. Sinton, D. P. Weitekamp, A. Pines, Selective excitation of multiple-quantum coherence in nuclear magnetic resonance. *Phys. Rev. Lett.* **43**, 1791–1794 (1979).

34. A. J. Menssen, A. E. Jones, B. J. Metcalf, M. C. Tichy, S. Barz, W. S. Kolthammer, I. A. Walmsley, Distinguishability and many-particle interference. *Phys. Rev. Lett.* **118**, 153603 (2017).

35. A. E. Jones, A. J. Menssen, H. M. Chrzanowski, T. A. W. Wolterink, V. S. Shchesnovich, I. A. Walmsley, Multiparticle interference of pairwise distinguishable photons. *Phys. Rev. Lett.* **125**, 123603 (2020).

36. V. S. Shchesnovich, M. E. O. Bezerra, Collective phases of identical particles interfering on linear multiports. *Phys. Rev. A* **98**, 033805 (2018).

37. D. A. Rice, C. F. Osborne, P. Lloyd, Multiparticle interference. *Phys. Lett. A* **186**, 21–28 (1994).

38. S. Agne, T. Kauten, J. Jin, E. Meyer-Scott, J. Z. Salvail, D. R. Hamel, K. J. Resch, G. Weihs, T. Jennewein, Observation of genuine three-photon interference. *Phys. Rev. Lett.* **118**, 153602 (2017).

39. T. Brünner, G. Dufour, A. Rodríguez, A. Buchleitner, Signatures of indistinguishability in bosonic many-body dynamics. *Phys. Rev. Lett.* **120**, 210401 (2018).

40. G. Dufour, T. Brünner, A. Rodríguez, A. Buchleitner, Many-body interference in bosonic dynamics. *New J. Phys.* **22**, 103006 (2020).

41. E. Brunner, “Many-body interference, partial distinguishability and entanglement,” thesis, Albert-Ludwigs-Universität Freiburg (2019).

42. A. M. Minke, A. Buchleitner, C. Dittel, Characterizing four-body indistinguishability via symmetries. *New J. Phys.* **23**, 073028 (2021).

43. J. J. Rotman, *The Theory of Groups: An Introduction* (Springer, 1995).

44. F. Graffitti, P. Barrow, M. Proietti, D. Kundys, A. Fedrizzi, Independent high-purity photons created in domain-engineered crystals. *Optica* **5**, 514–517 (2018).

45. A. Pickston, F. Graffitti, P. Barrow, C. L. Morrison, J. Ho, A. M. Brańczyk, A. Fedrizzi, Optimised domain-engineered crystals for pure telecom photon sources. *Opt. Express* **29**, 6991–7002 (2021).

46. A. Fedrizzi, A. Poppe, A. Zeilinger, T. Herbst, T. Jennewein, A wavelength-tunable fiber-coupled source of narrowband entangled photons. *Opt. Express* **15**, 15377–15386 (2007).

47. M. M. Weston, H. M. Chrzanowski, S. Wollmann, A. Boston, J. Ho, L. K. Shalm, V. B. Verma, M. S. Allman, S. W. Nam, R. B. Patel, S. Slussarenko, G. J. Pryde, Efficient and pure femtosecond-pulse-length source of polarization-entangled photons. *Opt. Express* **24**, 10869–10879 (2016).

48. S. Meraner, R. J. Chapman, S. Frick, R. Keil, M. Prilmüller, G. Weihs, Approaching the Tsirelson bound with a Sagnac source of polarization-entangled photons. *SciPost Phys.* **10**, 017 (2021).

49. R. Heilmann, J. Sperling, A. Perez-Leija, M. Gräfe, M. Heinrich, S. Nolte, W. Vogel, A. Szameit, Harnessing click detectors for the genuine characterization of light states. *Sci. Rep.* **6**, 19489 (2016).

50. D. Huber, M. Reindl, S. F. C. da Silva, C. Schimpf, J. Martín-Sánchez, H. Huang, G. Piredda, J. Edlinger, A. Rastelli, R. Trotta, Strain-tunable GaAs quantum dot: A nearly dephasing-free source of entangled photon pairs on demand. *Phys. Rev. Lett.* **121**, 033902 (2018).

51. F. Sbresny, L. Hanschke, E. Schöll, W. Rauhaus, B. Scaparra, K. Boos, E. Zubizarreta Casalengua, H. Riedl, E. del Valle, J. J. Finley, K. D. Jöns, K. Müller, Stimulated generation of indistinguishable single photons from a quantum ladder system. *Phys. Rev. Lett.* **128**, 093603 (2022).

52. Y. Karli, D. A. Vajner, F. Kappe, P. C. A. Hagen, L. M. Hansen, R. Schwarz, T. K. Bracht, C. Schimpf, S. F. Covre da Silva, P. Walther, A. Rastelli, V. M. Axt, J. C. Loredo, V. Remesh, T. Heindel, D. E. Reiter, G. Weihs, Controlling the photon number coherence of solid-state quantum light sources for quantum cryptography. *Npj Quantum Inf.* **10**, 17 (2024).

53. L. Zhai, G. N. Nguyen, C. Spinnler, J. Ritzmann, M. C. Löbl, A. D. Wieck, A. Ludwig, A. Javadi, R. J. Warburton, Quantum interference of identical photons from remote GaAs quantum dots. *Nat. Nanotechnol.* **17**, 829–833 (2022).

54. T. B. Pittman, D. V. Strekalov, A. Migdall, M. H. Rubin, A. V. Sergienko, Y. H. Shih, Can two-photon interference be considered the interference of two photons? *Phys. Rev. Lett.* **77**, 1917–1920 (1996).

55. Y.-H. Kim, W. P. Grice, Quantum interference with distinguishable photons through indistinguishable pathways. *J. Opt. Soc. Am. B* **22**, 493–498 (2005).

56. D. Bouwmeester, J.-W. Pan, M. Daniell, H. Weinfurter, A. Zeilinger, Observation of three-photon greenberger-horne-zeilinger entanglement. *Phys. Rev. Lett.* **82**, 1345–1349 (1999).

57. M. Hillery, V. Bužek, A. Berthiaume, Quantum secret sharing. *Phys. Rev. A* **59**, 1829–1834 (1999).

58. G. Murta, F. Grasselli, H. Kampermann, D. Bruß, Quantum conference key agreement: A review. *Adv. Quantum Technol.* **3**, 2000025 (2020).

59. G. S. Thekkadath, M. E. Mycroft, B. A. Bell, C. G. Wade, A. Eckstein, D. S. Phillips, R. B. Patel, A. Buraczewski, A. E. Lita, T. Gerrits, S. W. Nam, M. Stobińska, A. I. Lvovsky, I. A. Walmsley, Quantum-enhanced interferometry with large heralded photon-number states. *Npj Quantum Inf.* **6**, 89 (2020).

60. M. C. Tichy, Sampling of partially distinguishable bosons and the relation to the multidimensional permanent. *Phys. Rev. A* **91**, 022316 (2015).

61. R. Jozsa, Fidelity for mixed quantum states. *J. Mod. Opt.* **41**, 2315–2323 (1994).

62. J. Altepeter, E. Jeffrey, P. Kwiat, in *Advances in Atomic, Molecular, and Optical Physics* (Academic Press, 2005), vol. 52, pp. 105–159.

63. C. Dittel, "About the interference of many particles," thesis, University of Innsbruck (2019).

64. R.-B. Jin, M. Fujiwara, T. Yamashita, S. Miki, H. Terai, Z. Wang, K. Wakui, R. Shimizu, M. Sasaki, Efficient detection of an ultra-bright single-photon source using superconducting nanowire single-photon detectors. *Opt. Commun.* **336**, 47–54 (2015).

65. Cube beamsplitter, non-polarizing, 12.7 mm, 1550 nm laser line (2023); www.newport.com/p/05BC16NP.11.
